# Systematic investigation of chemo-immunotherapy synergism to shift anti-PD-1 resistance in cancer

**DOI:** 10.21203/rs.3.rs-3290264/v1

**Published:** 2023-09-14

**Authors:** Yue Wang, Dhamotharan Pattarayan, Haozhe Huang, Yueshan Zhao, Sihan Li, Yifei Wang, Min Zhang, Song Li, Da Yang

**Affiliations:** 1 Center for Pharmacogenetics, Department of Pharmaceutical Sciences, University of Pittsburgh, PA 15261, USA; 2 UPMC Hillman Cancer Institute, University of Pittsburgh, Pittsburgh, PA 15261, USA; 3 Department of Computational and Systems Biology, University of Pittsburgh, Pittsburgh, PA, 15261, USA

## Abstract

Chemo-immunotherapy combinations have been regarded as one of the most practical ways to improve immunotherapy response in cancer patients. In this study, we integrated the transcriptomics data from immunotherapy-treated tumors and compound-treated cell lines to systematically identify chemo-immunotherapy synergisms and their underlying mechanisms. Through analyzing anti-PD-1 treatment induced expression changes in patient tumors, we developed a shift ability score that can measure whether a chemotherapy treatment shifts anti-PD-1 response. By applying the shift ability analysis on 41,321 compounds and 16,853 shRNA treated cancer cell line expression profiles, we characterized a systematic landscape of chemo-immunotherapy synergism and prioritized 17 potential synergy targets. Further investigation of the treatment induced transcriptomic data revealed that a mitophagy-dsRNA-MAVS-dependent activation of type I IFN signaling may be a novel mechanism for chemo-immunotherapy synergism. Our study represents the first comprehensive effort to mechanistically characterize chemo-immunotherapy synergism and will facilitate future pre-clinical and clinical studies.

## INTRODUCTION

Immune checkpoint blockades, which block the inhibitory checkpoints and restore the cancer immune response, have significantly improved patient prognosis in several cancer types such as melanoma ^1,2^, lung cancer ^3^, colorectal cancer ^4^, and triple-negative breast cancer ^5^. However, immunotherapy is still not available for majority of cancer patients. Studies have shown that the immunotherapy response rate in melanoma patients ranges from 20%–30% ^1,2^. In other cancer types, such as breast, prostate, and colon cancers, the immunotherapy response rates range from 13% to 38% ^6^. Even for the patients who initially respond to the therapy, the later developed immunotherapy resistance remains to be challenging. There is an urgent need to identify effective strategies to overcome immunotherapy resistance and improve the overall response rate.

Emerging studies have reported that some chemo- and targeted therapy agents can induce significant effects on immune response in tumors ^7^. For example, gemcitabine is a synthetic pyrimidine nucleoside analogue which has been widely used as standard-of-care treatments in various cancers ^8,9^. Gemcitabine can induce immunogenic cell death, which enhance the dendritic cell-dependent cross-presentation of tumor antigens to cytotoxic T cells ^10,11^. Of note, by 2023, FDA have approved several chemo-immunotherapy regimens in diffuse large B-cell lymphoma (Polatuzumab + bendamustine/rituximab), triple-negative breast cancer (Atezolizumab/Pembrolizumab + taxanes), gastric cancer and esophageal adenocarcinoma (Nivolumab + FU−/platinum). As more chemo-immunotherapy combination regimens are being investigated and validated by ongoing clinical trials ^12,13^, they are becoming one of the most feasible paths to obtaining durable, long-lasting immunotherapy responses.

However, the design of the combination regimens so far is largely relied on clinical experiences, it is very challenging to characterize new chemo-immunotherapy synergisms. The emerging large-scale pharmacological transcriptomic datasets that profile the expression changes after drug/immunotherapy treatment provide us deeper and novel insights on how treatment changes biological processes in the tumor. These data present us an excellent opportunity to computationally model the interaction between chemotherapy and immunotherapy.

In current study, we hypothesize that the treatment-induced gene expression changes in tumor could be utilized to determine the immunotherapy outcome and to reveal the underlying resistance mechanism. Using anti-PD-1 induced expression changes, we characterized gene signatures that can robustly predict immunotherapy responses in patients. Importantly, we demonstrated that genetic inhibition of these signature genes can shift the immunotherapy response phenotypes. With these observations, we developed shift ability score to quantify a treatment’s capability of improving anti-PD-1 response. Through in silico screening on 41,321 compound-treated and 16,853 shRNA-treated cell line expression profiles, we identified known and novel treatments that can potentially shift anti-PD-1 resistance. Finally, we revealed that mitophagy-dsRNA-MAVS-dependent activation of type I IFN signaling may be a novel mechanism for chemo-immunotherapy synergism.

## RESULTS

### Treatment-induced expression change profiling can predict anti-PD-1 response in patients.

**Fig. 1:**
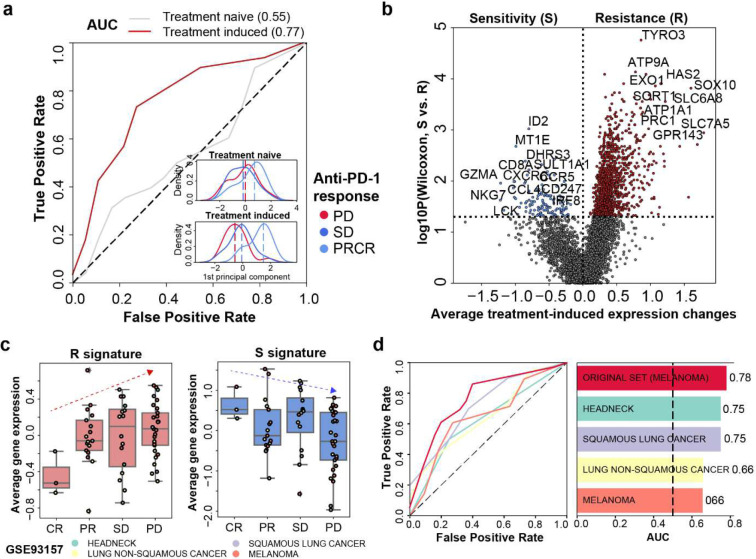
Treatment-induced expression changes can predict anti-PD-1 response in patients. **a**, Receiver operating characteristic (ROC) curve showing the performance of using treatment-naïve (grey) or treatment-induced (red) expression to classify anti-PD-1 responders and non-responders. The kernel density estimation plot in the corner showed the distribution of patient response groups on the first principal component of treatment-naïve expression (upper) or treatment-induced expression (lower). **b**, Volcano plot showing the identification of R and S signatures. Y-axis represents the Wilcoxon rank-sum test of gene expression on treatment-induced level between anti-PD-1 sensitive patients and resistant patients. The highlighted genes have significantly differential treatment-induced change between response groups. Blue-highlighted genes have higher treatment-induced expression changes in anti-PD-1 sensitive patients (i.e., S signature). Red-highlighted genes have higher expression changes in anti-PD-1 resistant patients, (i.e., R signature). **c**, Average gene expression of R signature genes (left) and S signature genes (right) across different response group in multiple cancer types (GSE93157). CR: complete response. PR: partial response. SD: stable disease. PD: progressive disease. **d**, Integrating R and S signature to classify anti-PD-1 responders and non-responders in training cohort (GSE91061) and GSE93157. ROC curve (left) shows the classification performance in different cancer types. Bar plot (right) shows the area under the curve (auc) of the corresponding ROC curve on the left panel.

We obtained the paired transcriptomic data from 68 melanoma patients before and after immunotherapy (i.e., nivolumab)^14^ (Extended Data Fig. 1a, b). This data allowed us to calculate the anti-PD-1-induced expression changes for each patient. Further principal component analysis (PCA) revealed that the anti-PD-1-induced expression achieved a better outcome classification performance (AUC=0.77) than treatment-naïve expression alone (AUC=0.55) (Fig. 1a, b and Extended Data Fig. 1b).

With this observation, we sought to characterize genes that showed the most differential anti-PD-1-induced expression changes between responders and non-responders. This analysis identified 1,190 and 130 genes as anti-PD-1 resistance (R) and sensitivity (S) signature, respectively (Fig. 1c and Supplementary Table 1). The S and R signatures’ ability in predicting anti-PD-1 response were further validated in three independent cohorts (GSE93157, GSE168204 and PHS001919) (Fig. 1c, d and Extended Fig. 1c to i). In those patients, the improved immunotherapy outcome was negatively correlated with the expression of R signature genes (p = 0.007, Student’s t test) and was positive correlated with that of S signature genes (p = 0.05, Student’s t test) (Fig. 1c and Extended Data Fig. 1d).

Further combining the gene expression of R signature and S signature can robustly classify responders from non-responders in patients with head and neck cancer (AUC= 0.75), squamous lung cancer (AUC=0.75), non-squamous lung cancer (AUC=0.66), and melanoma (AUC=0.66) (Fig. 1d, Extended Data Fig. 1e, g and Supplementary Table 1). Together, these results suggest that R and S signatures, which is based on treatment-induced expression changes, can readily predict immunotherapy response in multiple cancer types.

### Genes involved in R and S signatures are highly correlated with patient prognosis and immune responses.

**Fig. 2:**
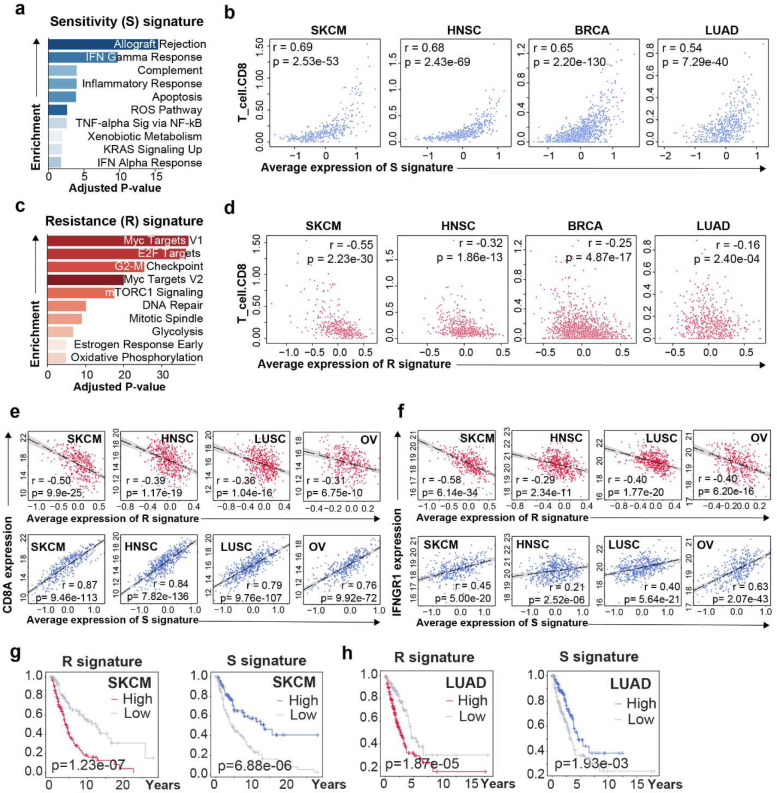
R and S signature associate with anti-tumor immunity in cancer patients. **a**, Pathway enrichment of genes involved in S signature. X-axis represents adjusted P-value derived from gene set enrichment analysis. Color degree represents the enrichment score derived from the same analysis. **b**, Scatter plots showing the association between CD8^+^ T cell infiltration and average S gene expression across TCGA cancer types. **c**, Pathway enrichment of genes involved in R signature. X-axis represents adjusted P-value derived from gene set enrichment analysis. Color degree represents the enrichment score derived from the same analysis. **d**, Scatter plots showing the association between CD8^+^ T cell infiltration and average S gene expression across TCGA cancer types. **e** and **f**, Scatter plots showing the association between CD8A e, or IFNGR1 (**f**) expression (log2FPKM-UQ) and average R (upper) or S (lower) gene expression across TCGA cancer types. **g** and **h**, Kaplan-Meier plots of patients grouped by average R (**g**) or S (**h**) gene expression in melanoma (upper) and lung adenocarcinoma (lower). High (low) groups are defined as top (bottom) one-third average expression in the corresponding cancer types.

Pathway analysis revealed that S signature genes are highly enriched in anti-cancer immune pathways (Fig. 2a). In 9,626 patients across 23 cancer types from TCGA database, we observed a dramatically negative correlation between expression of R genes and S genes (rho=−0.50, p=0.0, spearman’s correlation) (Extended Data Fig. 2a). Specifically, we observed that S signature expression are strongly correlated with immune-hot phenotypes such as the infiltration of CD8^+^ T cells (rho=0.69, melanoma), CD4^+^ T cells (rho=0.55, melanoma), dendritic cells (rho=0.85, melanoma), and other immune cells (Fig. 2b, Extended Data Fig. 2b and Supplementary Table 2).

On the other hand, R signature genes are mostly involved in immune evasion and cancer progression (Fig. 2c and Supplementary Table 2). Increased expression of R signature is highly correlated with decreased infiltration of CD8^+^ T cells (rho=−0.55), CD4^+^ T cells (rho=−0.58), macrophages (rho=−0.55), dendritic cells (rho=−0.68) and neutrophils (rho=−0.69) in melanoma, lung cancer, breast cancer, and stomach cancer (Fig. 2d, Extended Data Fig. 2b and Supplementary Table 2).

This observation is further supported by the negative correlation between R genes and the expression of CD8A (rho=−0.50, melanoma) and IFNGR1 (rho=−0.58, melanoma) (Fig. 2e), as well as the positive correlation between S genes and the expression of CD8A (rho=0.87, melanoma) and IFNGR1 (rho=0.45, melanoma) in multiple cancer types (Fig. 2f).

Consistent with their strong correlation with anti-cancer immune response, R genes show strong associations with poor prognosis in multiple cancer types. These cancer types include melanoma (HR=3.26, p=0.01, overall survival), lung cancer (HR=7.87, p<0.005), head and neck carcinoma (HR=7.29, p<0.005), and liver cancer (HR=9.72, p<0.005) (Fig. 2g, h, and Extended Data Fig. 2c, d). In contrast, high expression of S genes is significantly correlated with better prognosis in melanoma (HR=0.83, p=0.04), breast cancer (HR=0.72, p=0.03), and liver cancer (HR=0.65, p=0.01) (Fig. 2g, h, Extended Data Fig. 2c, e, and Supplementary Table 2).

### Genetic inhibition of genes in R and S signature can shift immunotherapy response phenotypes.

**Fig. 3:**
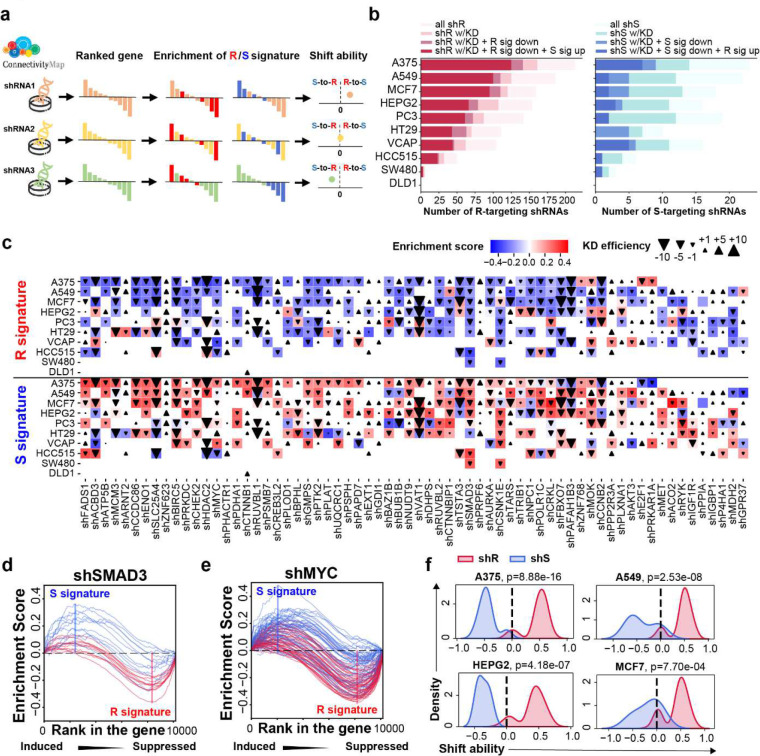
Genetic inhibition of genes in R and S signature can shift immunotherapy response phenotypes. **a**, Graph demonstration of R and S signature enrichment and shift ability analysis. **b**, Number of R (left) or S (right) targeting shRNAs that are able to knock down the target genes (shX w/KO), to suppress the target signature (X sig down), and to induce the other signature while suppressing the target signature (X sig down + Y sig up). **c**, Suppression of R signature (above) and induction of S signature (bottom) by shRNAs targeting the R signature genes. Color scale indicates the enrichment score of corresponding signatures in each experiment. Size of triangles indicates the knockdown efficiency given by the expression changes of target genes compared to other experiments in the same panel. Direction of triangles indicates the direction of expression changes. **d**, Enrichment curves of R signature and S signature in SMAD3 knockdown cell lines. **e**, Enrichment curves of R signature and S signature in MYC knockdown cell lines. **f**, Distribution of shift ability score of shRNAs targeting R signatures (shR) or S signatures (shS) across different cell lines. P values are given by two sample KS test.

To further determine if targeting R and S gene is enough to reverse immunotherapy outcome, we integrated the post-shRNA-treatment transcriptomes from Connectivity Map 2020 (CMAP2020) ^15^(Fig. 3a). In total, there are 454 genes involved in R or S signatures that have been targeted by shRNAs in 10 cancer cell lines across 6 cancer types (Supplementary Table 3). Among the 405 genes in R signature, 346 genes can be successfully knockdown by shRNA in at least one cell line (Fig. 3b and Extended Data Fig. 3a). When looking into the expression changes induced by shRNA, 92.2% (319 out of 346) R-gene targeted shRNAs can suppress the overall R signature expression (Supplementary Table 3) in at least one cell line. Notably, 91.5% (292 out of 319) of them can significantly upregulate the S signature expression in the same cells after genetic knockdown (Fig. 3b, c). For example, in A375 melanoma cell line, we observed the successful knockdown of R genes can significantly induce the expression of S genes (p=6.38e-89, paired t test) (Fig. 3c and Extended Data Fig. 3a). This observation could be expanded to other cancer cell lines including A549 (lung cancer), MCF7 (breast cancer), HEPG2 (liver cancer), PC3 (prostate cancer) and HT29 (colon-rectal cancer) (Fig. 3c and Extended Data Fig. 3b). On the other hand, among the 49 genes in S signature, 37 genes can be successfully knockdown by shRNA in at least one cell line (Supplementary Table 3). Similar to our observation in R signature, 59.4% (22 out of 37) of S-gene targeted shRNAs can lead to the overall suppression of S signature expression in at least one cell line (Extended Data Fig. 3c), and 16 (72.7%) of them can activate R signature expression in the same cells (Fig. 3b, c, and Extended Data Fig. 3d).

These observations suggest that genes in R and S signatures can not only predict, but also regulate the anti-PD-1 response. For example, SMAD3 is one of the R signature genes that are dramatically induced in anti-PD-1 resistant patients (Extended Data Fig. 3e). shRNA knockdown of the SMAD3 strongly inhibits the R signature while activating S signature gene expression (Fig. 3d). Previous studies have shown that SMAD3 plays an important role in tumor immune response by modulating MHC ^16,17^, interferon signaling ^18^ and NFKB pathway ^19^. The inhibition of TGFβ-SMAD3 signaling can sensitize immunotherapy response in melanoma ^20^ and pancreatic cancer ^21^. Besides SMAD3, our analysis recapitulated many established master regulators of tumor immune response, including MYC ^22^ and HDAC2 ^23^ (Fig. 3c, e, and Extended Data Fig. 3f).

Given the genetic perturbation of genes in R and S signatures can shift tumor response to anti-PD-1 treatment, we designed a shift ability score. The shift ability score summarizes the treatment’s overall capacity in inhibiting R signature and inducing S signatures and thus can quantify the treatment’s potential to shift immunotherapy outcomes (see **Methods**). As shown in cell lines including A375 (melanoma), A549 (lung cancer), HEPG2 (liver cancer) and MCF7 (breast cancer), genetic inhibition of R genes can lead to a resistant-to-sensitive shifting (R-to-S shifting), whereas in most of the cases, the genetic inhibition of S genes lead to a sensitive-to-resistant shifting (S-to-R shifting) (Fig. 3f and Extended Data Fig. 3g).

### Shift ability analysis on compound-treated transcriptomes characterized chemo-immunotherapy synergism.

We next sought to utilize the shift ability analysis to systematically characterize the compounds that can be synergistic with anti-PD-1 treatment. We collected 41,321 post-treatment transcriptome profiles across 64 cell lines from CMAP2020 database. These cell lines were treated by 4,264 compounds targeting 392 pathways at different dosages. We evaluated the shift ability of each treatment experiment (i.e., one compound tested in one cell line at one specific dosage). In total, we have identified 780 R-to-S shifting compounds that showed significant R-to-S shift (shift ability >= 0.7) in at least one treatment experiment (Extended Data Fig. 4a and Supplementary Table 4).

**Fig. 4:**
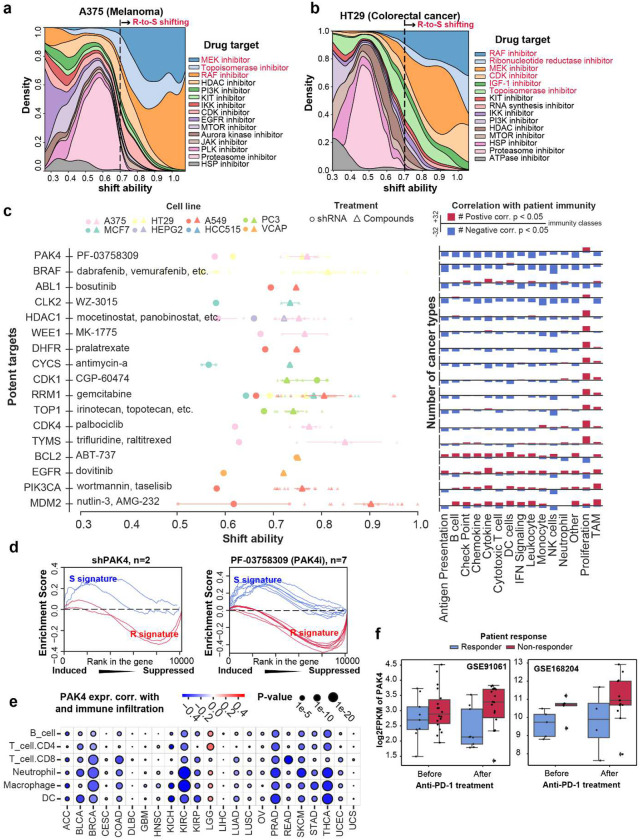
Shift ability analysis on compound-treated transcriptomes identified the landscape of chemo-immunotherapy synergism. **a**, Stacked density plot of top R-to-S shifting drug targets in A375 melanoma cell line. X-axis indicates shift ability. Y-axis indicates density. Red-highlighted text indicates the major drug targets in significant R-to-S shifting range (shift ability >= 0.7). **b**, Stacked density plot of top R-to-S shifting drug targets in HT29 colorectal cell line. X-axis indicates shift ability. Y-axis indicates density. Red-highlighted text indicates the major drug targets in significant R-to-S shifting range (shift ability >= 0.7). **c**, Prioritized potent targets for chemo-immunotherapy synergism. Drug names showed beside the potent gene targets are their corresponding pharmacological inhibitors. Circles indicate the shift ability of shRNAs, with big circles showing the average and small circles showing the individual experiments. Triangles indicate the shift ability of compound treatment, with big triangles showing the average and small triangles showing the individual experiments. Bar plots on the right side of the strip plot showed the number of TCGA cancer types where the corresponding genes have significantly positive (red) or negative (blue) correlation with different anti-tumor immunity signatures. **d**, Enrichment curves of R signature and S signature in PAK4 knockdown (left) and PAK4 inhibitor treated (right) cell lines (A375 and HT29). **e**, Association between PAK4 expression and immune cell infiltration in TCGA samples. **f**, PAK4 expression in patients before and after anti-PD-1 therapy from cohort GSE91061 and cohort GSE168204.

Some of the identified compounds activate the R-to-S shift in a cancer-specific manner (Fig. 4a, b, and Extended Data Fig. 4b to g). This observation is especially prominent for targeted therapy compounds. For example, MEK inhibitors are ranked high for R-to-S shifting ability in A375, HT29, A549, HELA and YAPC cells (Fig. 4a, b, and Extended Data Fig. 4b, f and g). This is consistent with the clinical indication that MEK signaling is activated in melanoma, colorectal cancer, non-small cell lung cancer, ovarian cancer, and pancreatic cancer ^24–26^. The estrogen receptor antagonist, on the other hand, showed significant R-to-S shifting ability exclusively in ER^+^ cell lines (MCF7 and VCAP), indicating that its shift ability is relied upon cell lines’ expression of estrogen receptor (Extended Data Fig. 4c, h). Another example is that EGFR inhibitors can induce significantly higher R-to-S shifting in EGFR expressing HCC515 compared to other cell lines (Extended Data Fig. 4h) ^27^. These results suggest that, for those targeted therapy compounds, the induction of the R-to-S shift is still relied on the availability of drug targets.

In this regard, we performed the shift ability analysis on additional genetic knockdown (i.e., shRNA treatment) of 300 drug targets across 10 cell lines (Supplementary Table 5). Combining the compound and shRNA screening result revealed 49 drug targets whose genetic and pharmacological inhibition can induce R-to-S shifting in the same cell lines. We have further evaluated the association between these drug targets and anti-tumor immunity in patient samples across 32 TCGA cancer types (see Methods). This analysis leads to a prioritized list of 17 drug targets whose inhibition showed strong association with immune activation in patient tumors (Fig. 4c and Supplementary Table 5).

The prioritized drug targets not only included previously reported chemoimmunotherapy synergisms, such as BRAF ^28^, RRM1 ^10,11^, CDK1^29^, CDK4^30^, HDAC1^23^, TOP1 ^31–33^, but also a couple of novel targets whose capability of regulating immune response have not been studied until recent. For example, we identified PAK4 as top potent target of chemoimmunotherapy synergism. Specifically, both genetic knockdown and pharmacological inhibition of PAK4 showed drastic R-to-S shift ability in A375 (melanoma) and HT29 (colorectal cancer) (Fig. 4c, d). For cell lines in which only PAK4 inhibitor (i.e., PF-03758309) data are available, we also observed significant R-to-S shift ability induced by PAKi in MCF7 (breast cancer), PC3 (prostate cancer) and HCC515 (lung cancer) (Extended Data Fig. 4i to k).

In patient tumor samples, PAK4’s inhibition is positively correlated with immune-hot tumor microenvironment in 22 cancer types. Among which breast cancer, kidney cancer, prostate cancer, melanoma, and colorectal cancer showed the most significant correlation (Fig. 4e and Extended Data Fig. 5l to n). In two anti-PD-1 treated patient cohorts (GSE91061 and GSE168204), PAK4 expression significantly increased in non-responders. Interestingly, this difference is much greater in post-treatment profiling compared to pre-treatment baseline, reinforcing the importance of including post-treatment profiling in identifying key regulators of anti-PD-1 response (Fig. 4f).

Notably, recent studies have shown that pharmacological inhibition of PAK4 is a very promising therapy to be combined with immunotherapies. This includes compound PF-03758309, which improve CAR-T therapy in glioblastoma ^34^, as well as compound KPT-9274, which has been shown to improve anti-PD-1 response in melanoma ^35^. Together, our study successfully recapitulated the PAK4 as a promising drug target to boost immunotherapy efficacy.

**Fig. 5:**
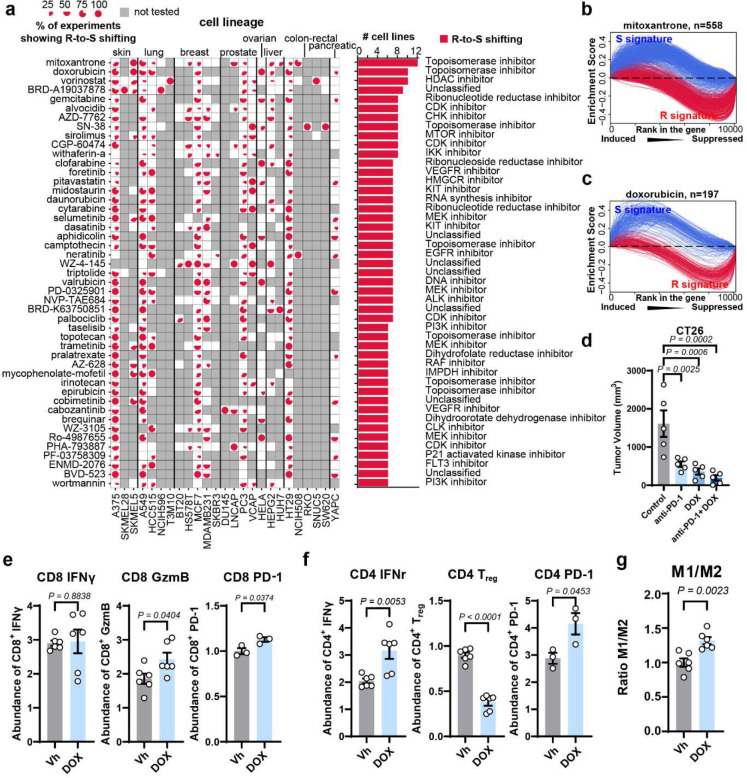
Landscape of pan-cancer chemo-immunotherapy synergism. **a**, Compounds that showed R-to-S shifting in multiple cell lines. Pie charts in each cell indicate the percentage of experiments showed a R-to-S shift ability. The bar plot on the right side of the pie matrix indicates the number of cell lines where the compounds showed R-to-S shifting in at least one experiment. Untested cell lines are shaded by grey. **b**, Enrichment curves of R signature and S signature in mitoxantrone treated cell lines. **c**, Enrichment curves of R signature and S signature in doxorubicin treated cell lines. **d**, Tumor volumes in CT26 tumor bearing mice after anti-PD-1, doxorubicin and combination treatments. n = 6 mice per group. **e**, Single-cell suspensions were prepared from CT26 tumor samples and subjected to flow cytometry analysis of CD4^+^ subtype T cells (CD4^+^ IFNγ^+^ T cells, FoxP3^+^ T cells, CD4^+^ PD-1^+^ T cells). **f**, Single-cell suspensions were prepared from CT26 tumor samples and subjected to flow cytometry analysis of CD8^+^ subtype T cells (CD8^+^ IFNγ^+^ T cells, GzmB^+^ T cells, CD8^+^ PD-1^+^ T cells). **g**, M1/M2 ratio of tumor associated macrophages (TAM) in CT26 colorectal tumor tissues

In addition to the cancer-type specific targeted therapy, we also observed that many drugs were predicted to increase the anti-PD-1 response in “pan-cancer” style (Fig. 5a and Extended Data Fig. 5a, b). For example, mitoxantrone, a topoisomerase inhibitor, showed significant R-to-S shifting ability in 11 cell lines across multiple cancer types (Fig. 5a, b). This observation is consistent with previous studies that mitoxantrone can induce immunogenic cell death, which will activate type I interferon signaling and facilitate the MHC-II-mediated antigen presentation through dendritic cells ^31–33^. Other topoisomerase inhibitors, e.g., doxorubicin (Fig. 5a, c), camptothecin, topotecan, irinotecan and epirubicin (Fig. 5a and Supplementary Table 4), also showed R-to-S shifting potential in multiple cell lines.

To validate our prediction, we established CT26 syngeneic mouse model to test the synergism between doxorubicin and anti-PD-1 therapy in vivo. We observed that the suppression on tumor growth by the combination therapy is significantly improved compared to anti-PD-1 or doxorubicin treatment alone (Fig. 5d). Co-treatment of anti-PD-1 and doxorubicin can remarkably decrease tumor volume in 18 days treatment (p = 0.0002). Particularly, doxorubicin treatment can significantly increase CD8^+^ GzmB^+^, CD8^+^ PD-1^+^, CD4^+^ IFN-γ ^+^, and CD4^+^ PD-1^+^ T cell populations in tumor microenvironment (Fig. 5e, f, and Extended Data Fig. 5c to e), as well as M1/M2 ratio (Fig. 5g and Extended Data Fig. 5g). These results demonstrated that doxorubicin treatment can active tumor immune response and is synergistic with anti-PD-1.

### Mechanistic analysis revealed that the treatment-induced mitophagy may be a novel mechanism for chemo-immunotherapy synergism.

**Fig. 6:**
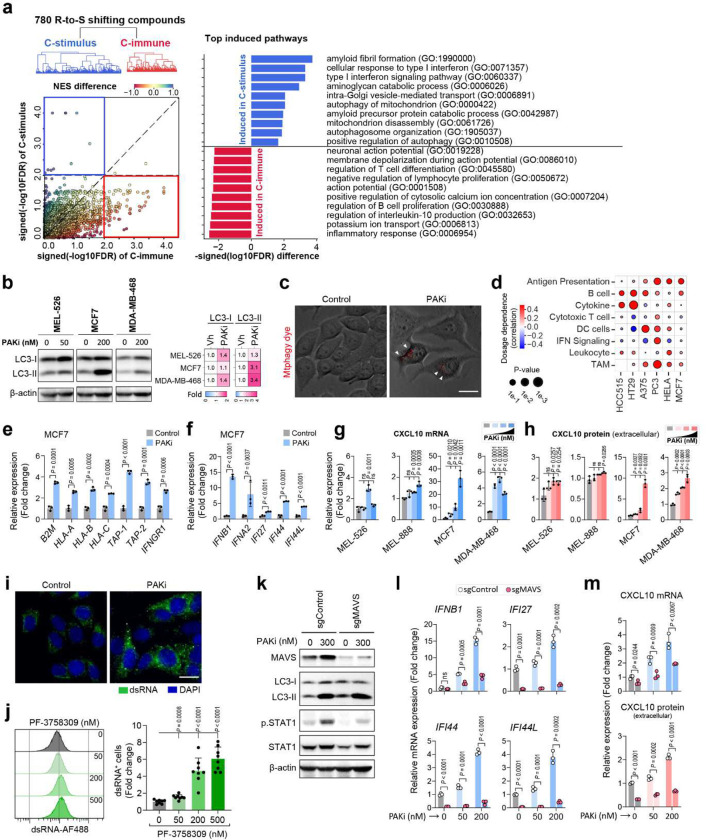
PAK4 inhibitor can induce immune response through autophagy-mtRNA-MAVS-CXCL10 axis in cancer cells. **a,** Treatment induced expression analyses reveals mechanisms of chemo-immunotherapy synergisms. **b**, Immunoblotting analysis of LC3 protein in cancer cells after 48 h of PF-03758309 (PAKi) treatment. Heat map (Right) indicates fold change of LC3-I and LC3-II band intensity, normalized to respective β-actin. **c**, PAKi treatment induces mitophagy in MCF7 cells. Florescence assay-based detection of mitophagy in 24 h DMSO and 500 nM PF-03758309 treated MCF7 cells. Arrowheads indicate mtphagy dye signals, Scale bar: 20 μm. **d**, Association between dosage and immunity induction of PAK4 inhibitor PF-03758309 in multiple cancer cell lines. PF-03758309 induces mitophagy in MCF7 cells. MCF7 cells were treated with 500 nM of PF-03758309 **e** and **f**, qRT-PCR validation of antigen presenting and processing genes (**e**) and interferon stimulated genes (**f**) in MCF7 cells after 48 h of PAKi (200 nM) treatment. 0 nM or vehicle served as control, *n* = 3 technical replicates. **g** and **h**, The CXCL10 expression, detected by qRT-PCR (**g**) and ELISA analysis (h), in cancer cells after 48 h of PAKi treatment. Concentration of PF-03758309 used: 0, 2, 50 and 500 nM for MEL-526; 0, 50, 200 and 500 nM for MEL-888, MCF7 and MDA-MB-468 cells. *n* = 3 technical or biological replicates. **i**, PAKi treatment induces dsRNA accumulation in MCF7 cells. Immunofluorescence analysis in 24 h DMSO or PF-03758309 treated MCF7 cells. Scale bar: 20 μm. **j**, PAKi treatment induces dose-depended dsRNA expression in MCF7 cells (24 h). *n* = 8 replicates from two independent experiments with 4 biological replicates. **k**, Immunoblotting analysis in MCF7 sgControl and sgMAVS cells after 48 h of PAKi treatment. **l**, qRT-PCR analysis of *IFNB1* and interferon stimulated genes in MCF7 sgControl and sgMAVS cells after 48 h of PAKi treatment. *n* = 3 technical replicates. **m**, The CXCL10 expression, detected by qRT-PCR (top) and ELISA analysis (bottom), in MCF7 sgControl and sgMAVS after 48 h of PAKi treatment. *n* = 3 technical or biological replicates. Data in **e-h**, **j**, **l**, and **m** are presented as mean ± SD, *P* values were generated using a two-tailed Student’s t-test, *P* > 0.05 was indicated as ns.

We next sought to systematically characterize the potential mechanism for the synergism between anti-PD-1 and R-to-S shifting compounds. Overall, 780 R-to-S shifting compounds can be clustered into two groups based on treatment-induced changes of different molecular processes (Fig. 6a). The most common activated molecular processes from each cluster revealed that one major cluster (“C-immune”) showed a direct induction of immune response (NES=2.25, FDR=0.0028). The compound in this cluster appears to be able to direct induce the genes involved in antigen presentation and immune cell recruitment ^36^. In contrast, the other major cluster (“C-stimulus”) exhibited a significant induction of type I interferon (NES=2.42, FDR<1e-4), suggesting the compounds triggered some stimulus, which further activate the interferon pathways (Fig. 6a).

Notably, most compounds in cluster “C-stimulus” seems to induce autophagy related processes, including the mitophagy (autophagy of mitochondrion, NES=2.13, FDR=0.0017). Mitophagy is an essential way that tumor cells rely on to deal with the damaged mitochondria and protect themselves from chemotherapy-induced cell death ^37^. Recently, mitochondrial DNA (mtDNA) and RNA (mtRNA) released during mitophagy have been characterized as novel triggers of tumor-intrinsic immune response ^38,39^. Of note, our shift ability analysis revealed multiple MEK inhibitors, such as selumetinib and PD-0325901 can induce dramatic R-to-S shifting in melanoma, lung adenocarcinoma, colorectal cancer, and breast cancer cell lines (Supplementary Table 6). These MEK inhibitors are enriched in “C-stimulus” cluster and significantly induced mitophagy and CXCL10 expression. Consistent with our prediction, a recent study demonstrated that MEK inhibitors can induce mitophagy, which lead the release of mtDNA and activation of TLR9-dependent CXCL10 production^40^.

### PAKi induced mitophagy-dsRNA-MAVS-dependent activation of type I IFN signaling in tumor.

In addition to MEK inhibitors, we identified that PAK inhibitor (PAKi) is also categorized to “C-stimulus” cluster. Although PAKi have been shown to have strong synergism with anti-PD-1 by multiple studies ^34,35^, the underlying mechanism remains elusive. Our analysis revealed that PAKi, PF-03758309, can strongly activate mitophagy (Extended Data Fig. 6a). To validate our computational analysis, we treated MCF-7, MEL-526 and MDA-MB-468 cells with PF-03758309 and observed that the PF-03758309 treatment can increase LC3-I and LC3-II protein levels^41,42^ (Fig. 6b and Supplementary Table 5). The increased expression of LC3-I and LC3-II protein are indicators of autophagosomes accumulation and mitophagy. Further mitophagy staining assay confirmed that PAKi treatment can increase mitophagy in MCF7 cells (Fig. 6c).

As a potential result of treatment induced mitophagy, cells treated by PF-03758309 showed upregulated antigen presentation in multiple cancer cell lines (Fig. 6d,e, Extended Data Fig. 6b and Supplementary Table 7). Moreover, we also observed the PF-03758309 induced mitophagy upregulates type I interferon signaling (Fig. 6f, Extended Data Fig. 6c,d and Supplementary Table 8), CXCL10 expression (Fig. 6g,h), as well as *PD-L1* expression (Extended Data Fig. 6e).

We next sought to determine if PAKi-induced mitophagy activates mtDNA-STING pathway^40^ to enhance anti-tumor immune response. Surprisingly, in MEL-526, MCF7 and MDA-MB-468 cells, the PF-03758309 treatment does not lead to STING expression (i.e., total STING) or activation (i.e., phosphorylated STING) (Extended Data Fig. 6f, g). Besides, enzymatic DNA depletion cannot rescue PF-03758309-induced activation of type I interferon signaling (Extended Data Fig. 6h, i), suggesting that mtDNA does not mediate PAKi’s activation of immune response.

Alternatively, mitophagy will also lead to the release of mitochondrial RNA (mtRNA), which may trigger dsRNA sensors and activate type I IFN pathway^43^. Indeed, we observed increased cytoplasmic accumulation and a significant dose-dependent expression of dsRNA after PAKi treatment (Fig. 6i,j). To further demonstrate if PAKi induced immune activation is mediated by dsRNA signaling, we knocked out cytosolic dsRNA sensor MAVS in MCF7 cells (sgMAVS). The MAVS knockout significantly abolished type I interferon signaling, CXCL10, and antigen presenting genes expression induced by PAKi (Fig. 6k–m, and Extended Data Fig. 6j). Together, our data suggest that PAKi-induced mitophagy and consequent mtRNA release is required for PAKi to activate the type I interferon pathway and immune response.

## DISCUSSION

In this study, we have established the treatment-induced gene signatures for anti-PD-1 response. The signatures’ predictive performance is robust across multiple independent patient cohorts. Genes in the anti-PD-1 resistance (R) and sensitivity (S) signatures are highly associated with anti-tumor immune response and patient prognosis across different cancer types. Most importantly, our analyses on shRNA-treated transcriptomic data demonstrated that the signature genes can not only predict but also regulate anti-PD-1 response.

These discoveries enlightened us to conceptualize the shift ability score and screen 4,264 chemo-/targeted therapy compounds in multiple cancer types. By further integrating with the genetic knockdown screening, we identified 17 drug targets whose pharmacological and genetic inhibition exhibit consistent immunotherapy shift ability. Moreover, our study also characterized and experimentally validated FDA approved cancer drugs, such as doxorubicin, is synergetic with anti-PD-1 therapy. We expect these discoveries can be quickly translated to patient care and have an impact on cancer therapy.

The current study has limitations. Since the post-treatment profiling of compounds and shRNAs are mostly available in cancer cell lines, our prediction focused on characterizing the compound/drug’s impact on immunotherapy response that are mediated by tumor cell-intrinsic mechanisms. The tumor cell-intrinsic mechanisms have been recently revealed to play important roles in immunotherapy response^44^. Numerous studies have demonstrated that quite some FDA approved chemotherapies achieve therapeutic effect by making tumor cells more recognizable or attractive to the immune system in addition to directly killing tumor cells^13^. Most of drugs that are FDA-proved to be combined with immunotherapy are working through increasing tumor cell antigen presentation, immunogenetic cell death, and secretion of the cytokine^45^.

In this study, the focus of the drug induced cancer cell transcriptomic data helped us to build a landscape on how drugs/compounds regulate cell-intrinsic mechanisms and eventually influence immunotherapy response. Our study revealed two major mechanisms for the established chemo-immunotherapy synergisms. We found that some drugs, including FDA approved CDK inhibitors, can induce genes involved in the direct regulation of immune response. Other drugs, such gemcitabine, topoisomerase inhibitors, and MEK inhibitors, induce genes related to interferon response and autophagy. We also validated a novel PAK4 inhibitor PF-03758309 to induce type I interferon signaling and CXCL10 secretion in tumor through activation of mitophagy-mediated mtRNA-MAVS signaling.

While mitophagy has been studied as one major mechanism of chemo-resistance in cancer ^37^, its role in anti-tumor immunity has not been fully appreciated until recently ^46–48^. The mitophagy-mediated release of mtDNA and mtRNAs activates the anti-viral signaling, which will initiate the innate immune response and induce the CXCL10 secretion ^40^. Both innate immune response and CXCL10 secretion have been demonstrated to increase the efficacy of immune checkpoint blockades ^49^. Future clinical studies are warranted to determine whether drug-induced mitophagy should be minimized to control chemo-resistance or be exploited to synergize immunotherapy.

Collectively, our study characterized a comprehensive landscape for chemo-immunotherapy synergism, which will facilitate the ongoing efforts on designing novel chemo-immunotherapy combinations and improve the patient’s overall prognosis.

## MATERIALS AND METHODS

### Data collection and preprocessing

Post-perturbation cell line transcriptome data, including shRNA and compound treatment, were collected from the Expanded Connectivity Map (CMap) LINCS Resource 2020 (complete version, 11/23/2021) through the CLUE portal (http://clue.io). The analyses were based on CMap level 5 signature matrices, with 238,351×12,328 in dimension for shRNA perturbation and 720,216×12,328 for compound treatment. Since the level 5 signatures were constructed based on replicates, only signatures with sufficient transcriptional activity score (>=0.4) were retained for further analyses to reduce the false signals introduced by low reproducibility. Since CMap is based on L1000 panel which detected 978 landmark genes and then inferred the rest ten thousand genes based on the landmarks, we only utilized the expression information of 9,196 best-inferred genes together with the landmark genes (10,174 genes in total). These filtering procedures led to the final matrix of 16,853×10,174 for shRNA perturbation and 41,321×10,174 for compound treatment.

Gene expression and clinical data of patients treated with immune checkpoint blockade were collected from Gene Expression Omnibus (GEO) with accession number GSE91061, GSE168204 and GSE93157 (https://www.ncbi.nlm.nih.gov/geo/). Among them, GSE91061 had 43 pairs of melanoma samples (n=86) pre- and on-treatment. According to the original publication, 18 pairs with progressive disease (PD) were considered as non-responders; 15 pairs with stable disease (SD) and 9 pairs with partial/complete response (PRCR) were considered as responders; 1 pair of samples with unknown (UNK) response was excluded from the analysis. GSE168204 had 27 unpaired melanoma samples pre- (n=10) and post-treatment (n=17), of which 18 were non-responders and 9 were responders. 3 samples were excluded because of too high or too low overall expression: MGH530_FFPE_032417 (post, R), MGH27_070213 (post, NR) and 148-12-13-14_S10 (post, NR). GSE93157 had 65 pre-treatment samples from melanoma (n=25), lung non-squamous cancer (n=22), lung squamous lung cancer (n=13) and head and neck cancer (n=5). According to the original publication, these samples were further divided into 4 response groups: PD (progressive disease, n=29), SD (stable disease, n=16), PR (partial response, n=17), and CR (complete response, n=3). All these three data were mapped to the same gene space as CMAP2020, leading to the final dimension of 84×10,157 for GSE91061, for 24×10,059 for GSE168204 and 65×766 for GSE93157.

Gene expression and clinical data of The Cancer Genome Atlas (TCGA) patients were obtained from the GDC data portal (http://portal.gdc.cancer.gov). The analyses in this study were restricted to primary tumors except for melanoma where metastatic samples were focused, resulting in a total number of 9626 bulk tumor samples. To evaluate the immunity contents, we used TIMER ^50^ to estimate the infiltration abundance of cytotoxic T cells, B cells, dendritic cells, macrophages, and nature killer cells in each bulk tumor sample.

### Predicting anti-PD-1 response using patient gene expression profiles

To evaluate the ability of using different gene expression profiles to predict anti-PD-1 response in patients, we applied principal component analysis (PCA) on treatment-naïve expression and treatment-induced expression of nivolumab-treated patients from GSE91061, respectively. For each patient, we defined the response score as the first principal component of treatment-naïve/-induced expression profile, based on which patients are classified as non-responders and responders to nivolumab treatment. The classification performance was evaluated through receiver operating characteristic curve by comparing to the response groups defined in the original publication.

### Construction of anti-PD-1 response signature based on treatment-induced expression.

The anti-PD-1 response signature was constructed based on GSE91061, where paired samples were collected pre- and on-nivolumab treatment. For each paired sample, the treatment-induced expression change of a gene is defined as the log2-transformed fold change between on-treatment and pre-treatment expression. Wilcoxon rank-sum test was applied to identify the most significant expression changes between responders and non-responders. Response signature for anti-PD-1 sensitive (S signature) was characterized as 139 genes whose treatment-induced expression changes were significantly (p < 0.05) higher in responders, whereas response signature for anti-PD-1 resistance (R signature) consisted of 1,190 genes whose treatment-induced expression changes were significantly higher (p < 0.05) in non-responders.

To investigate the function of genes involved in R and S signatures, gene set enrichment analysis was performed using cancer hallmarks from MSigDB. The significant enrichment was defined as an adjusted P-value lower than 0.05. To validate the ability of R and S signatures in recapitulating anti-PD-1 responses of patients from independent datasets, we utilized the gene expression data and treatment information of patient samples from the processed GSE168204 and GE93157. Since both of the datasets were not available for pre-on paired assessment, we used relative expressions differing from the cohort population baseline as a surrogate measurement of treatment-induced expression for each gene. Average expression changes of genes from R and S signature were then used to classify the patient response. The classification performance was evaluated through receiver operating characteristic curve by comparing to the response groups defined in publications.

### Prognosis and immunity assessment of R and S signature genes

To functionally annotate R and S signature, pathway enrichment analysis on cancer hallmarks is conducted by Gene Set Enrichment Analysis (GSEA). To demonstrate the immunity and prognosis relevance of R and S signature genes in a broader range of cancers, we investigated how R and S signature genes can indicate immune cell infiltration and survival outcome in TCGA patient samples. Although TCGA patients did not receive immunotherapy, they have undergone intrinsic immune response processes which lead to the infiltration of immune cells into the tumor microenvironments. To this end, for patients from each cancer type, we used relative gene expressions differing from the population baseline as a surrogate measurement of expression changes. Average expression changes of genes from R/S signature were then compared to the TIMER estimation of immune cell fractions for each cancer type. For survival analysis, Cox Proportional Hazard model was applied on progression free interval (PFI) with the average expression changes of R/S signature genes. For selected cancer types, patients were segregated into three groups evenly based on the average expression changes of R/S signature genes, and the log rank test was applied to the highest R/S group and the lowest R/S group accordingly.

### Enrichment assessment of R and S signature and the calculation of shift ability score

To assess whether expression of R or S signature genes can be changed by a given perturbation p(pϵ{shRNA,compound}), we utilized the enrichment score calculation by pre-rank Gene Set Enrichment Analysis^51^ with the weighting parameter set to 1. Specifically, for each perturbation p, a descending ranked gene list of size N, which contains treatment-induced expression changes of N genes $\left\{g_{1},g_{2},\ldots ,g_{N}\right\}$, is constructed according to CMap level 5 signature. Enrichment of R signature and S signature will then be calculated through pre-rank GSEA and termed as $ES_{R}$ and $ES_{S}$, respectively.

To evaluate the potential a given perturbation p(pϵ{shRNA,compound}) can shift a cell line to an anti-PD-1 sensitive state, we created the concept of “shift ability”. Briefly, the shift ability analysis will quantify the ability of a given perturbation in suppressing the R signature and inducing the S signature in a cell line. The shift ability score is thus given by the deviation from $ES_{S}$ of $ES_{R}$:

$${\mathrm{shift}\;\mathrm{ability}}_{p}\;=\Delta ES=ES_{S}-ES_{R}$$

A high, positive shift ability means the perturbagen p is able to suppress the R signature and at the same time promote the S signature, shifting the cell line to an immune-active and anti-PD-1 sensitive state. In contrast, a negative shift ability means the perturbation will potentially cause immune-suppression and anti-PD-1 resistance. Shift ability close to zero indicates the perturbagen might not be able to induce considerable shifting in immune response or have less effect on the immunotherapy efficacy.

### Immunity association of potent synergy targets

To evaluate the association between potent synergy targets and anti-tumor immunity in patients, we first collected 68 immune response gene signatures from previous studies ^52^. An enrichment score was calculated for each signature using the single-sample gene set enrichment (ssGSEA) analysis ^53^ for each patient from TCGA cohorts. Enrichment scores of immune response signatures from the same immunity class would be averaged. Pearson’s correlation was used to assess the association between patient immune response and potent synergy targets across different cancer types. An association with a p-value less than 0.05 would be considered as a significant correlation.

### Characterization of mechanism of chemo-immunotherapy synergism

3,983 post-perturbation expression profiles (level 5 signature) of 780 R-to-S shifting compounds across different cell lines were extracted. For each compound, a consensus gene expression change signature will be calculated using the following method: for each gene across the samples from the experiments of the same drug, if its treatment-induced expression is higher than 1, the expression indicator will set as 1; if its treatment-induced expression is lower than −1, the expression indicator will set as −1; otherwise, the expression indicator will set as 0. For each compound, the sum of expression indicators across the samples will be used as its consensus expression change level. Pearson correlations were calculated and were utilized as distance metric between compounds in the gene space of 9,196 best-inferred genes together with the landmark genes (10,174 genes in total). Based on the correlation matrix, hierarchical clustering analysis was performed using Ward method. Two major clusters were identified through the dendrogram cutoff at 12. The median value of the 10,174 genes’ consensus expression changes will be ranked by descending and used as the consensus gene expression change signature of that corresponding cluster. For mechanism annotation of the major clusters, pre-rank GSEA was performed to assess the pathway enrichment in consensus gene expression change vectors using 3019 GO terms with 1000 times of permutation.

### General Statistical analyses

For difference comparison, if not being particularly specified, Wilcoxon rank-sum test was applied to compare the differences between two unpaired groups; Wilcoxon signed rank test was applied to compare the differences between two paired groups; one-way ANOVA was applied to compare the differences between three or more groups; Kolmogorov-Smirnov test was applied to compare the differences between two continuous distributions. For correlation analysis, both Pearson and Spearman’s correlation were applied in order to avoid the potential conflicts on linearity assumption. For enrichment and exclusiveness, pre-rank Gene Set Enrichment Analysis was applied to assess the enrichment of specific features in single samples; hypergeometric test was applied for between-group comparison. For survival analysis, both Cox Proportional Hazard model and log rank test were utilized to compare prognosis between groups. All the computational and statistical analyses presented in this study were implemented by Python (version 3.8.0) in local or on the cluster of University of Pittsburgh Center for Research Computing (CRC).

### Animals

In vivo antitumor efficacy was tested in syngeneic mouse colon (CT26) cancer models. Female BALB/c mice (4–6 weeks) were subcutaneously (s.c.) inoculated with CT26 cells (5×10^5^ cells per mouse). When the tumor volume reached ~ 50 mm^3^, mice were randomly divided into two groups (n = 5) and treated via tail vein injection with PBS (control), DOX-loaded nanoparticles, respectively once every three days for three times (DOX: 5 mg/kg). Tumor sizes were monitored every three days following the initiation of the treatment and calculated by the formula: (Length × Width^2^)/2.

To evaluate the synergistic effects of anti-PD-1 and FASA/DOX, a syngeneic CT26 colon tumor model was established by inoculating 5×10^5^ CT26 cells into the flank of BALB/c mice. When the tumor volume reached ~100 mm^3^, mice were randomly grouped (n = 5), and treated with PBS (control), PD-1 antibody (BioCell), nanoparticles loaded DOX, and anti-PD-1+nanoparticles loaded DOX, respectively, every three days for a total of three times (DOX: 2.5 mg/kg, anti-PD-1: 5 mg/kg). Tumor volumes were monitored every three days and calculated as described above.

### Cell lines and reagents

Human melanoma cancer cell lines MEL-526 and MEL-888, human breast cancer cell lines MCF7 and MDA-MB-468 were purchased from American Type Culture Collection (ATCC). MEL-526 and MEL-888 cells were cultured in RPMI-1640 (Hyclone) supplemented with 10% heat-inactivated Fetal Bovine Serum (HI-FBS) (Gibco). MCF7 and MDA-MB-468 cells were cultured in Dulbecco’s Modified Eagle Medium (DMEM) (Hyclone). All cells were cultured with presence of 1X Penicillin-Streptomycin during normal growth conditions. During the drug treatments cells were cultured with antibiotic-free growth media. The PAK inhibitor PF-3758309 (PAKi) purchased from Selleckchem (Cat. #S7094). The drug was dissolved in dimethyl sulfoxide (DMSO). Cell lines were treated with 0 to 500 nM of PAKi for 48 h. In nucleic acid depletion assay cells were treated with 10 U/mL of DNase or 10 U/mL of RNase (Invitrogen).

### Antibodies

The following antibodies were used for immunoblotting analysis: rabbit anti-LC3A/B (CST, #12741), (rabbit anti-STAT1 (CST, #14994, rabbit anti-phospho-STAT1 (CST, #7649), rabbit anti-STING (CST, #13647), rabbit anti-phospho-STING (CST, #72971), rabbit anti-MAVS (CST, #3393), and mouse anti-β-actin (Sigma-Aldrich, #A5441). Goat anti-mouse and Goat anti-mouse IgG HRP conjugated secondary antibodies (SCBT, #SC-2005, #SC-2004).

### RNA isolation and quantitative Real-Time Polymerase Chain Reaction (qRT-PCR) Assay

Total RNA from cells were isolated using TRIzol (ThermoFisher, # 15596018) as per the manufacturer’s instructions. Following RNA isolation, 1–2 μg of total RNAs used to synthesize cDNAs using High-Capacity cDNA Reverse Transcription Kit (Applied Biosystems, #4368813). qPCR analyses were performed using *Power* SYBR^™^ Green PCR Master Mix (Applied Biosystems, #4367659) on QuantStudio 6 Pro Real-Time PCR System (Applied Biosystems). Relative mRNA expressions were determined by calculating ΔΔCt values normalized to *GAPDH*, two-tailed Student’s t-test used to calculate statistical values. Sequences of primers used for qRT-PCR are listed below.

**Table:** 

Target	Forward (5’ --> 3’)	Reverse (5’ --> 3’)
*GAPDH*	GGTGAAGGTCGGAGTCAACG	TGGGTGGAATCATATTGGAACA
*B2M*	ATGTCTCGCTCCGTGGCCTT	GACTTTCCATTCTCTGCTGG
*HLA-A*	AAAAGGAGGGAGTTACACTCAGG	GCTGTGAGGGACACATCAGAG
*HLA-B*	CTACCCTGCGGAGATCA	ACAGCCAGGCCAGCAACA
*HLA-C*	CACACCTCTCCTTTGTGACTTCAA	CCACCTCCTCACATTATGCTAACA
*TAP-1*	GCTGTTCCTGGTCCTGGTGG	TTTCGAGTGAAGGTATCGGC
*TAP-2*	CAATAGCAGCGGAGAAGGTG	CTCGGCCCCAAAACTGCGAA
*IFNGR1*	GTCAGAGTTAAAGCCAGGGTTG	CTTCCTGCTCGTCTCCATTTAC
*PD-L1*	CTACTGGCATTTGCTGAACG	GACAATTAGTGCAGCCAGGT
*CXCL10*	GCTGCCTTATCTTTCTGACT	GGACAAAATTGGCTTGCAGG
*IFNB1*	CTTGGATTCCTACAAAGAAGCAGC	TCCTCCTTCTGGAACTGCTGCA
*IFNA2*	TGGGCTGTGATCTGCCTCAAAC	CAGCCTTTTGGAACTGGTTGCC
*IFI27*	CGTCCTCCATAGCAGCCAAGAT	ACCCAATGGAGCCCAGGATGAA
*IFI44*	GTGAGGTCTGTTTTCCAAGGGC	CGGCAGGTATTTGCCATCTTTCC
*IFI44L*	TGCACTGAGGCAGATGCTGCG	TCATTGCGGCACACCAGTACAG
*MAVS*	ATGGTGCTCACCAAGGTGTCTG	TCTCAGAGCTGCTGTCTAGCCA
*TRAF6*	CAATGCCAGCGTCCCTTCCAAA	CCAAAGGACAGTTCTGGTCATGG

### Immunoblotting

Total proteins from specified cells extracted using RIPA lysis buffer contains 1X protease and phosphatase inhibitor. Protein concentration of each sample measured using BCA protein assay kit (ThermoFisher, #23227) as per the manufacturer’s instructions. Then, equal concentration of proteins from each sample were taken and mixed appropriate volume of 5X protein sample buffer supplemented with reducing agent. Subsequent incubation at 98°C for 10 min, equal amount of each protein sample was subjected to SDS-polyacrylamide gel electrophoresis using 4–12% gel and transferred to polyvinylidene difluoride (PVDF) membrane (Bio-Rad, #162–0177). The protein transferred membranes were immunoblotted with appropriate primary antibodies for overnight at 4°C followed by appropriate horseradish peroxidase (HRP) conjugated secondary antibodies for 1 h at room temperature. Signal was visualized by enhanced chemiluminescence substrate (ThermoFisher, #F32106) and exposed using iBright CL1500 imaging system (Invitrogen). Band intensities were quantified using ImageJ software and normalized to β-actin, heatmaps were generated using GraphPad Prism 9.5.1.

### Mitophagy assay

Mitophagy detection kit was purchased from Dojindo (Rockvile, MD). Cells were seeded on 96 well clear bottom black wall tissue culture plate. After 24 h, cells were washed twice with serum free medium followed by incubation with Mtphagy Dye at 37°C for 30 min. After two washed cells, cells were incubated with or without drugs for 24 h. After incubation cells washed twice with serum-free medium and images were obtained using KEYENCE BZ-X800 Fluorescence Microscope.

### Cloning, sgRNA Construction and Lentiviral Transduction

We designed three guide RNA (gRNA) for MAVS and one scrambled gRNA as a control and used lentiCRISPRv2 vector. Lentiviral particles were prepared after transfection of plasmids into HEK-293T cells using Lipofectamine 2000^™^ (Invitrogen, #11668019). Targeted cells were infected with the lentivirus packaged by Cas9 and single-guide RNA (sgRNA) expression plasmid encoding puromycin resistance (Addgene plasmid, #52961). The knockout efficiency was determined by the immunoblotting analysis of MAVS after selection of puromycin resistance cells. gMAVS_F3/R3 exhibited the maximum knockout efficiencies of MAVS gene in MCF7 cells and were used for subsequent experiments. Guide RNA sequences used to generate MAVS knockout:

gMAVS_F1: caccgCTTCCGGTCGGCTTGTGGCC;

gMAVS_R1: aaacGGCCACAAGCCGACCGGAAGc;

gMAVS_F2: caccgAGGTGGCCCGCAGTCGATCC;

gMAVS_R2: aaacGGATCGACTGCGGGCCACCTc;

gMAVS_F3: caccgGTGTCTTCCAGGATCGACTG;

gMAVS_R3: aaacCAGTCGATCCTGGAAGACACc

### Flowcytometry analysis

Flow cytometry was performed with LSRII (BD Biosciences) and Aurora (Cytek Biosciences) instruments and analyzed by FlowJo (BD Biosciences). CT26 tumors were collected 1 day after the last treatment. Single-cell suspensions were prepared. Briefly, tumors were dissected and transferred into RPMI-1640. Tumors were disrupted mechanically using scissors, digested with a mixture of deoxyribonuclease I (0.3 mg/ml, Sigma-Aldrich) and TL Liberase (0.25 mg/ml, Roche) in serum-free RPMI-1640 at 37 °C for 30 min, and dispersed through a 40 μm cell strainer (BD Biosciences). After red blood cell lysis, live/dead cell discrimination was performed using a Zombie NIR Fixable Viability Kit (BioLegend, dilution: 1/1,000) at 4 °C for 30 min in PBS. Surface staining was performed at 4 °C for 30 min in FACS staining buffer (1× phosphate-buffered saline/5% FBS/0.5% sodium azide) containing designated antibody cocktails (PerCP anti-mouse CD45 antibody, Brilliant Violet 785 anti-mouse CD4 antibody, Brilliant Violet 480 anti-mouse CD8 antibody, PE anti-mouse PD-1 antibody and APC/Cyanine7 anti-mouse F4/80 antibody; dilution: 1/200 for all antibodies). For intracellular protein staining (Pacific Blue anti-mouse Foxp3 antibody and FITC anti-mouse CD206 antibody; dilution: 1/200 for both antibodies), cells were fixed and permeabilized using the BD Cytofix/Cytoperm kit, following the manufacturer’s instructions. For intracellular cytokine staining (PE-Cy7 anti-mouse IFN-γ antibody and AF647 anti-mouse GzmB antibody; dilution: 1/200 for antibody), cells were stimulated with phorbol 12-myristate-13-acetate (100 ng/ml) and ionomycin (500 ng/ml) for 6 h in the presence of Monensin. Cells were fixed/permeabilized using the BD Cytofix/Cytoperm kit before cell staining. The numbers of immune cells normalized by the number of control group were presented as relative abundance of immune cells.

#### Flow cytometry analysis of dsRNA:

After 24 h treatment, cells were collected by trypsinization and washed twice with PBS. Cells were fixed with 4% formaldehyde for 20 min at room temperature (RT). After two washes using PBS, cells were permeabilized for 15 min at RT using 0.1% Triton X-100 in PBS. Cells then incubated with 1% BSA for 1 h at RT followed by incubation with 2.5 μg/mL of anti-dsRNA (J2) antibody (CST, #76651) for 1 h at RT. After three washed cells were incubated with 2.2 μg/mL of Alexa Fluor 488 conjugated goat anti-mouse IgG H&L antibody for 1 h at RT. Cells then washed three times with PBS and suspended in 0.5% BSA in PBS. Cells were analyzed using MACSQuant analyzer (Miltenyi Biotec). Data were analyzed using FlowJo Software (10.9.0).

## Supplementary Material

Supplement 1

## Data Availability

The source code in this study for shift ability analysis is available at https://github.com/dangoDANGO/SYNERGY. The authors declare that all the other scripts generating the figures and supporting the findings of this study are available from the corresponding author upon reasonable request. All the other data supporting the findings of this study are available in the article and its supplementary data files or from the corresponding author upon reasonable request. All data needed to evaluate the conclusions in the paper are present in the paper and/or the Supplementary Materials.

## References

[1] Ribas A. Association of Pembrolizumab With Tumor Response and Survival Among Patients With Advanced Melanoma. JAMA 315, 1600–1609, doi:10.1001/jama.2016.4059 (2016). 27092830

[2] Schadendorf D. Pooled Analysis of Long-Term Survival Data From Phase II and Phase III Trials of Ipilimumab in Unresectable or Metastatic Melanoma. J Clin Oncol 33, 1889–1894, doi:10.1200/JCO.2014.56.2736 (2015). 25667295 PMC5089162

[3] Brahmer J. Nivolumab versus Docetaxel in Advanced Squamous-Cell Non–Small-Cell Lung Cancer. New England Journal of Medicine 373, 123–135, doi:10.1056/NEJMoa1504627 (2015). 26028407 PMC4681400

[4] Smith K. M. & Desai J. Nivolumab for the treatment of colorectal cancer. Expert Review of Anticancer Therapy 18, 611–618, doi:10.1080/14737140.2018.1480942 (2018). 29792730

[5] Keenan T. E. & Tolaney S. M. Role of Immunotherapy in Triple-Negative Breast Cancer. Journal of the National Comprehensive Cancer Network J Natl Compr Canc Netw 18, 479–489, doi:10.6004/jnccn.2020.7554 (2020). 32259782

[6] Sharma P., Hu-Lieskovan S., Wargo J. A. & Ribas A. Primary, Adaptive, and Acquired Resistance to Cancer Immunotherapy. Cell 168, 707–723, doi:10.1016/j.cell.2017.01.017 (2017). 28187290 PMC5391692

[7] Zitvogel L., Apetoh L., Ghiringhelli F. & Kroemer G. Immunological aspects of cancer chemotherapy. Nat Rev Immunol 8, 59–73, doi:10.1038/nri2216 (2008). 18097448

[8] Noble S. & Goa K. L. Gemcitabine. A review of its pharmacology and clinical potential in non-small cell lung cancer and pancreatic cancer. Drugs 54, 447–472, doi:10.2165/00003495-199754030-00009 (1997). 9279506

[9] Gudena V., Montero A. J. & Gluck S. Gemcitabine and taxanes in metastatic breast cancer: a systematic review. Ther Clin Risk Manag 4, 1157–1164 (2008). 19337423 PMC2643097

[10] Nowak A. K. Induction of tumor cell apoptosis in vivo increases tumor antigen cross-presentation, cross-priming rather than cross-tolerizing host tumor-specific CD8 T cells. J Immunol 170, 4905–4913, doi:10.4049/jimmunol.170.10.4905 (2003). 12734333

[11] Nowak A. K., Robinson B. W. & Lake R. A. Synergy between chemotherapy and immunotherapy in the treatment of established murine solid tumors. Cancer Res 63, 4490–4496 (2003). 12907622

[12] Zhou X. Synergies of Antiangiogenic Therapy and Immune Checkpoint Blockade in Renal Cell Carcinoma: From Theoretical Background to Clinical Reality. Front Oncol 10, 1321, doi:10.3389/fonc.2020.01321 (2020). 32850419 PMC7403214

[13] Salas-Benito D. Paradigms on Immunotherapy Combinations with Chemotherapy. Cancer Discov 11, 1353–1367, doi:10.1158/2159-8290.CD-20-1312 (2021). 33712487

[14] Riaz N. Tumor and Microenvironment Evolution during Immunotherapy with Nivolumab. Cell 171, 934–949.e916, doi:10.1016/j.cell.2017.09.028 (2017). 29033130 PMC5685550

[15] Subramanian A. A Next Generation Connectivity Map: L1000 Platform and the First 1,000,000 Profiles. Cell 171, 1437–1452 e1417, doi:10.1016/j.cell.2017.10.049 (2017). 29195078 PMC5990023

[16] Dong Y., Tang L., Letterio J. J. & Benveniste E. N. The Smad3 Protein Is Involved in TGF-β Inhibition of Class II Transactivator and Class II MHC Expression1. The Journal of Immunology 167, 311–319, doi:10.4049/jimmunol.167.1.311 (2001). 11418665

[17] Berglund A. K., Long J. M. & Schnabel L. V. TGF-β downregulates MHC I surface expression through a Smad3-dependent mechanism. The Journal of Immunology 204, 140.142–140.142, doi:10.4049/jimmunol.204.Supp.140.2 (2020).

[18] Qing J. Transforming Growth Factor β/Smad3 Signaling Regulates IRF-7 Function and Transcriptional Activation of the Beta Interferon Promoter. Molecular and Cellular Biology 24, 1411–1425, doi:10.1128/MCB.24.3.1411-1425.2004 (2004). 14729983 PMC321430

[19] Mazi F. A. The paracaspase MALT1 is a downstream target of Smad3 and potentiates the crosstalk between TGF-β and NF-kB signaling pathways in cancer cells. Cellular Signalling 105, 110611, doi:10.1016/j.cellsig.2023.110611 (2023). 36708753

[20] Park J. Combination delivery of TGF-β inhibitor and IL-2 by nanoscale liposomal polymeric gels enhances tumour immunotherapy. Nature Materials 11, 895–905, doi:10.1038/nmat3355 (2012). 22797827 PMC3601683

[21] Peng H. Local Release of TGF-β Inhibitor Modulates Tumor-Associated Neutrophils and Enhances Pancreatic Cancer Response to Combined Irreversible Electroporation and Immunotherapy. Advanced Science 9, 2105240, doi:10.1002/advs.202105240 (2022). 35128843 PMC8981446

[22] Han H. Small-Molecule MYC Inhibitors Suppress Tumor Growth and Enhance Immunotherapy. Cancer Cell 36, 483–497.e415, doi:10.1016/j.ccell.2019.10.001 (2019). 31679823 PMC6939458

[23] Woods D. M. HDAC Inhibition Upregulates PD-1 Ligands in Melanoma and Augments Immunotherapy with PD-1 Blockade. Cancer Immunology Research 3, 1375–1385, doi:10.1158/2326-6066.CIR-15-0077-T (2015). 26297712 PMC4674300

[24] Rinn J. L. Functional demarcation of active and silent chromatin domains in human HOX loci by noncoding RNAs. Cell 129, 1311–1323, doi:10.1016/j.cell.2007.05.022 (2007). 17604720 PMC2084369

[25] Jänne P. A. Selumetinib plus docetaxel for KRAS-mutant advanced non-small-cell lung cancer: a randomised, multicentre, placebo-controlled, phase 2 study. The Lancet Oncology 14, 38–47, doi:10.1016/S1470-2045(12)70489-8 (2013). 23200175

[26] Van Cutsem E. Phase I/II trial of pimasertib plus gemcitabine in patients with metastatic pancreatic cancer. International Journal of Cancer 143, 2053–2064, doi:10.1002/ijc.31603 (2018). 29756206

[27] Gerber D. E. EGFR inhibition in the treatment of non-small cell lung cancer. Drug Development Research 69, 359–372, doi:10.1002/ddr.20268 (2008). 19562083 PMC2701650

[28] Liu L. The BRAF and MEK Inhibitors Dabrafenib and Trametinib: Effects on Immune Function and in Combination with Immunomodulatory Antibodies Targeting PD-1, PD-L1, and CTLA-4. Clinical Cancer Research 21, 1639–1651, doi:10.1158/1078-0432.CCR-14-2339 (2015). 25589619

[29] Hossain D. M. S. Dinaciclib induces immunogenic cell death and enhances anti-PD1–mediated tumor suppression. The Journal of Clinical Investigation 128, 644–654, doi:10.1172/JCI94586 (2018). 29337311 PMC5785250

[30] Zhang J. Cyclin D–CDK4 kinase destabilizes PD-L1 via cullin 3–SPOP to control cancer immune surveillance. Nature 553, 91–95, doi:10.1038/nature25015 (2018). 29160310 PMC5754234

[31] Mei K.-C. Liposomal Delivery of Mitoxantrone and a Cholesteryl Indoximod Prodrug Provides Effective Chemo-immunotherapy in Multiple Solid Tumors. ACS Nano 14, 13343–13366, doi:10.1021/acsnano.0c05194 (2020). 32940463 PMC8023019

[32] Li C. Mitoxantrone triggers immunogenic prostate cancer cell death via p53-dependent PERK expression. Cellular Oncology 43, 1099–1116, doi:10.1007/s13402-020-00544-2 (2020). PMC12990691 32710433

[33] Wang Q. Immunogenic cell death in anticancer chemotherapy and its impact on clinical studies. Cancer Letters 438, 17–23, doi:10.1016/j.canlet.2018.08.028 (2018). 30217563

[34] Ma W. Targeting PAK4 to reprogram the vascular microenvironment and improve CAR-T immunotherapy for glioblastoma. Nat Cancer 2, 83–97, doi:10.1038/s43018-020-00147-8 (2021). 35121889 PMC10097424

[35] Abril-Rodriguez G. PAK4 inhibition improves PD-1 blockade immunotherapy. Nat Cancer 1, 46–58, doi:10.1038/s43018-019-0003-0 (2020). 34368780 PMC8340852

[36] Banik D., Moufarrij S. & Villagra A. Immunoepigenetics Combination Therapies: An Overview of the Role of HDACs in Cancer Immunotherapy. International Journal of Molecular Sciences 20 (2019). 10.3390/ijms20092241 PMC6539010 31067680

[37] Vara-Perez M., Felipe-Abrio B. & Agostinis P. Mitophagy in Cancer: A Tale of Adaptation. Cells 8 (2019). 10.3390/cells8050493 PMC6562743 31121959

[38] Liu S., Feng M. & Guan W. Mitochondrial DNA sensing by STING signaling participates in inflammation, cancer and beyond. International Journal of Cancer 139, 736–741, doi:10.1002/ijc.30074 (2016). 26939583

[39] Grochowska J., Czerwinska J., Borowski L. S. & Szczesny R. J. Mitochondrial RNA, a new trigger of the innate immune system. WIREs RNA 13, e1690, doi:10.1002/wrna.1690 (2022). 34498404

[40] Limagne E. MEK inhibition overcomes chemoimmunotherapy resistance by inducing CXCL10 in cancer cells. Cancer Cell 40, 136–152.e112, doi:10.1016/j.ccell.2021.12.009 (2022). 35051357

[41] Chen W. Cold exposure alters lipid metabolism of skeletal muscle through HIF-1alpha-induced mitophagy. BMC Biol 21, 27, doi:10.1186/s12915-023-01514-4 (2023). 36750818 PMC9906913

[42] Strappazzon F. AMBRA1 is able to induce mitophagy via LC3 binding, regardless of PARKIN and p62/SQSTM1. Cell Death Differ 22, 419–432, doi:10.1038/cdd.2014.139 (2015). 25215947 PMC4326570

[43] Liu Q. Circulating mitochondrial DNA-triggered autophagy dysfunction via STING underlies sepsis-related acute lung injury. Cell Death & Disease 12, 673, doi:10.1038/s41419-021-03961-9 (2021). 34218252 PMC8254453

[44] Pitt J. M. Resistance Mechanisms to Immune-Checkpoint Blockade in Cancer: Tumor-Intrinsic and - Extrinsic Factors. Immunity 44, 1255–1269, doi:10.1016/j.immuni.2016.06.001 (2016). 27332730

[45] Yu W. D., Sun G., Li J., Xu J. & Wang X. Mechanisms and therapeutic potentials of cancer immunotherapy in combination with radiotherapy and/or chemotherapy. Cancer Lett 452, 66–70, doi:10.1016/j.canlet.2019.02.048 (2019). 30902563

[46] Zhong Z., Sanchez-Lopez E. & Karin M. Autophagy, Inflammation, and Immunity: A Troika Governing Cancer and Its Treatment. Cell 166, 288–298, doi:10.1016/j.cell.2016.05.051 (2016). 27419869 PMC4947210

[47] Jiang T., Chen X., Ren X., Yang J.-M. & Cheng Y. Emerging role of autophagy in anti-tumor immunity: Implications for the modulation of immunotherapy resistance. Drug Resistance Updates 56, 100752, doi:10.1016/j.drup.2021.100752 (2021). 33765484

[48] Jiang G.-M. The relationship between autophagy and the immune system and its applications for tumor immunotherapy. Molecular Cancer 18, 17, doi:10.1186/s12943-019-0944-z (2019). 30678689 PMC6345046

[49] Robin R. Immune cell and tumor cell-derived CXCL10 is indicative of immunotherapy response in metastatic melanoma. Journal for ImmunoTherapy of Cancer 9, e003521, doi:10.1136/jitc-2021-003521 (2021). 34593622 PMC8487215

[50] Li T. TIMER: A Web Server for Comprehensive Analysis of Tumor-Infiltrating Immune Cells. Cancer Research 77, e108–e110, doi:10.1158/0008-5472.CAN-17-0307 (2017). 29092952 PMC6042652

[51] Subramanian A. Gene set enrichment analysis: a knowledge-based approach for interpreting genome-wide expression profiles. Proc Natl Acad Sci U S A 102, 15545–15550, doi:10.1073/pnas.0506580102 (2005). 16199517 PMC1239896

[52] Thorsson V. The Immune Landscape of Cancer. Immunity 48, 812–830 e814, doi:10.1016/j.immuni.2018.03.023 (2018). 29628290 PMC5982584

[53] Barbie D. A. Systematic RNA interference reveals that oncogenic KRAS-driven cancers require TBK1. Nature 462, 108–112, doi:10.1038/nature08460 (2009). 19847166 PMC2783335

