# Supplementary material for: Systematic investigation of chemo-immunotherapy synergism to shift anti-PD-1 resistance in cancer
Source: Res Sq. 2023 Sep 14:rs.3.rs-3290264. Preprint. [Version 1] doi: 10.21203/rs.3.rs-3290264/v1 (PMC10543256; doi:10.21203/rs.3.rs-3290264/v1)
Supplement: Supplement 1 [file NIHPPrs3290264v1-supplement-1.pdf]

Figure S1

a

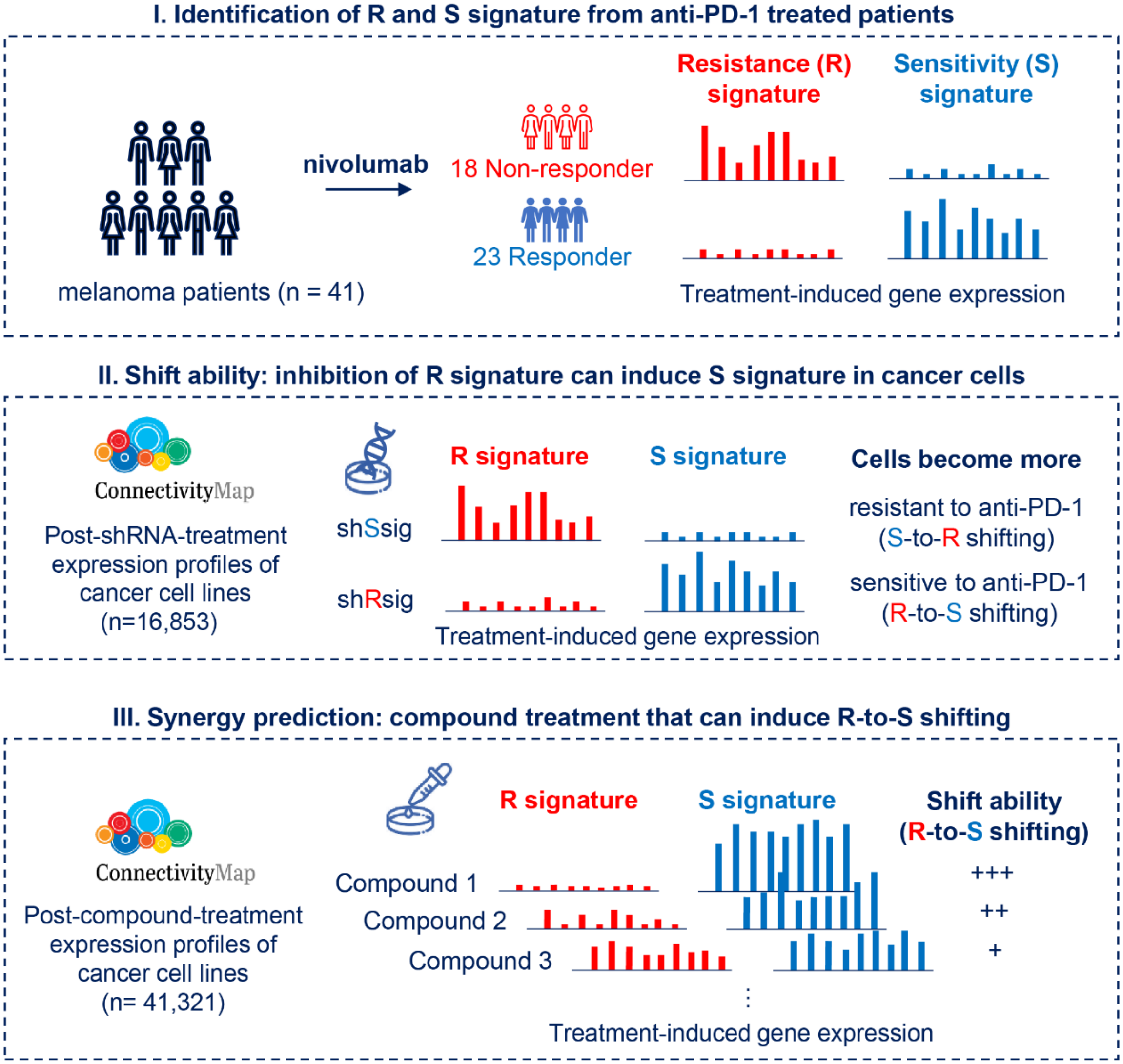

759

Figure S1 Cont'd

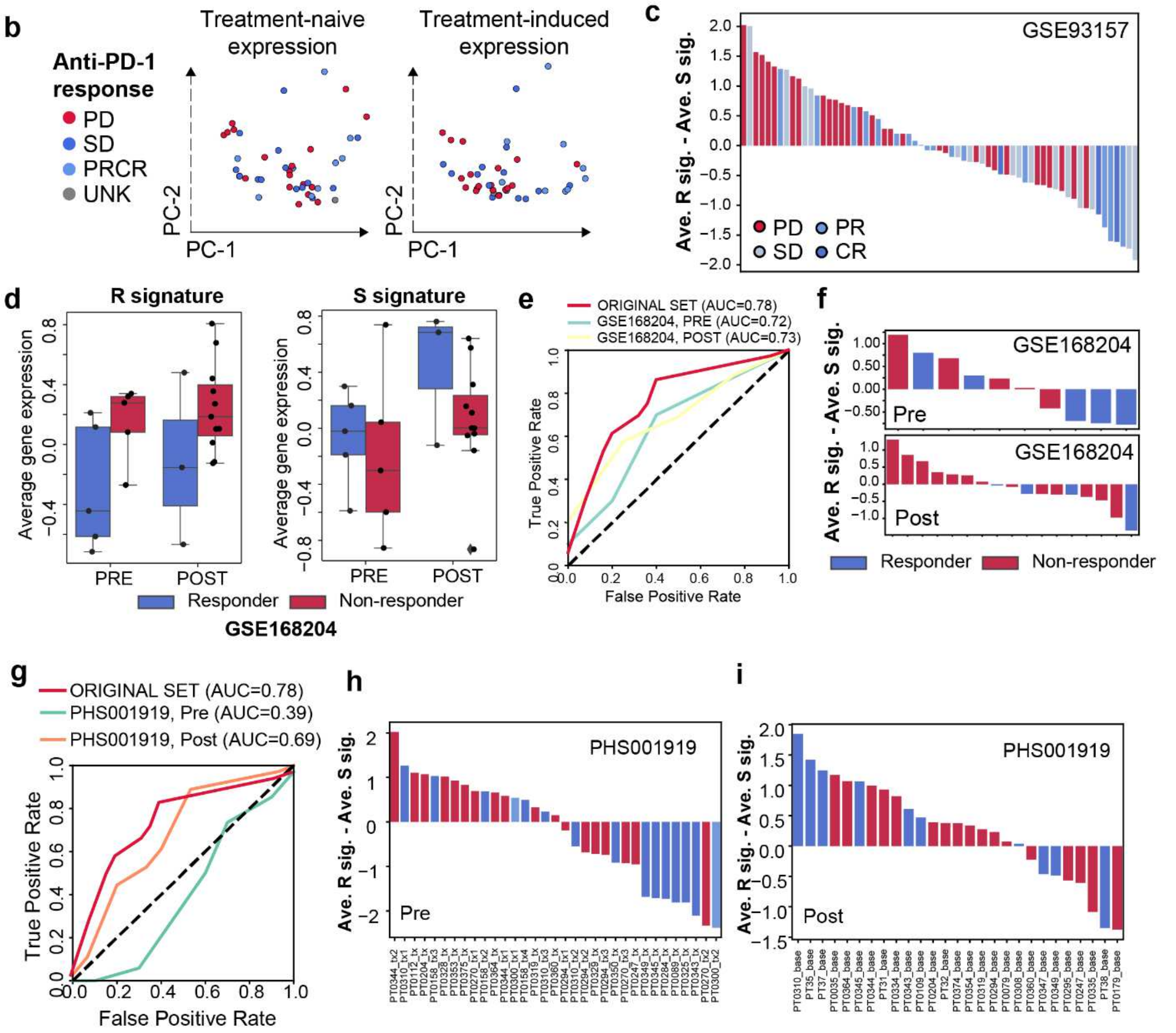

Extended Data Fig. 1 R signature and S signature genes are associated with anti-PD-1 and immune response in patients (Corresponding to Fig. 1)

a, Schematic of data analysis framework in this study.

b, Distribution of different response groups on the first principal component of treatment-naïve expression and treatment-induced expression.

c, Integrating R and S signature to classify anti-PD-1 responders and non-responders in patient cohort GSE93157. Patients are ranked in descending order based on the difference between R signature expression and S signature expression. Colors indicate the anti-PD-1 response group.

d, Average gene expression change of R signature genes (left) and S signature genes (right) in melanoma patients (GSE168204) before and after anti-PD-1 treatment.

e, Integrating R and S signature to classify anti-PD-1 responders and non-responders in original set GSE91061 and GSE1682604. ROC curve shows the classification performance in different time points.

f, Integrating R and S signature to classify anti-PD-1 responders and non-responders in GSE168204. Patients are ranked in descending order based on the difference between R signature expression and S signature expression. Colors indicate the anti-PD-1 response group.

g, Integrating R and S signature to classify anti-PD-1 responders and non-responders in original set GSE91061 and PHS001919. ROC curve shows the classification performance in different time points.

h, Integrating R and S signature to classify anti-PD-1 responders and non-responders in PHS001919. Patients (pre-treatment) are ranked in descending order based on the difference between R signature expression and S signature expression. Colors indicate the anti-PD-1 response group.

i, Integrating R and S signature to classify anti-PD-1 responders and non-responders in PHS001919. Patients (post-treatment) are ranked in descending order based on the difference between R signature expression and S signature expression. Colors indicate the anti-PD-1 response group.

Figure S2

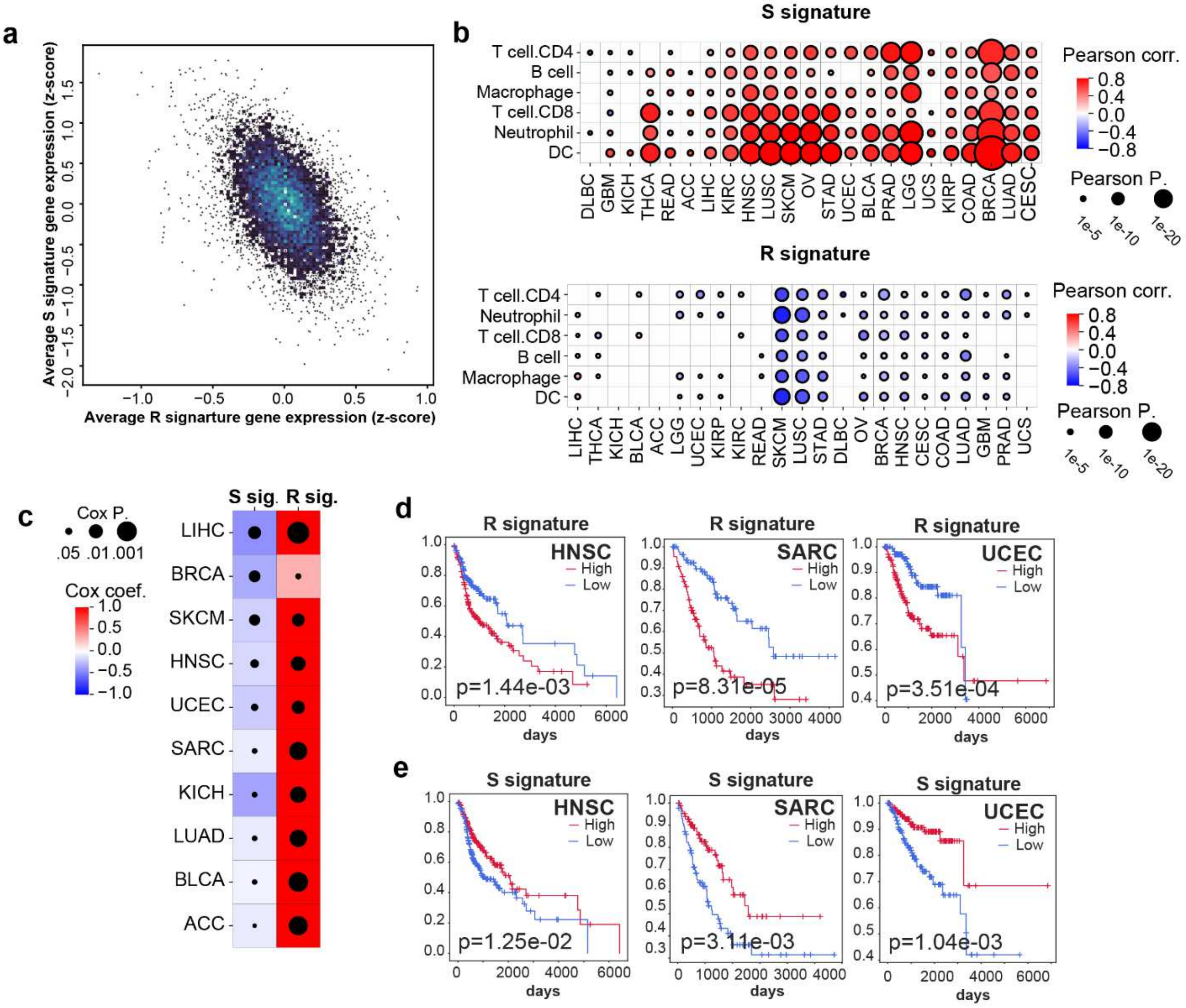

Extended Data Fig. 2 R and S signature associated with anti-tumor immunity in cancer patients. (Corresponding to Fig. 2)

a, Association between R signature expression and S signature expression in TCGA samples.

b, Association between S and R signature expression and immune infiltration in TCGA cohorts.

c, Cox regression showing the association between the R (S) signature expression and patient overall survival (OS) in TCGA cohorts.

d, Kaplan-Meier plots of patients grouped by average R gene expression in head and neck (left), sarcoma (middle) and endometrial cancer (right). High (low) groups are defined as top (bottom) one-third average expression in the corresponding cancer types.

e, Kaplan-Meier plots of patients grouped by average R gene expression in head and neck (left), sarcoma (middle) and endometrial cancer (right). High (low) groups are defined as top (bottom) one-third average expression in the corresponding cancer types.

Figure S3

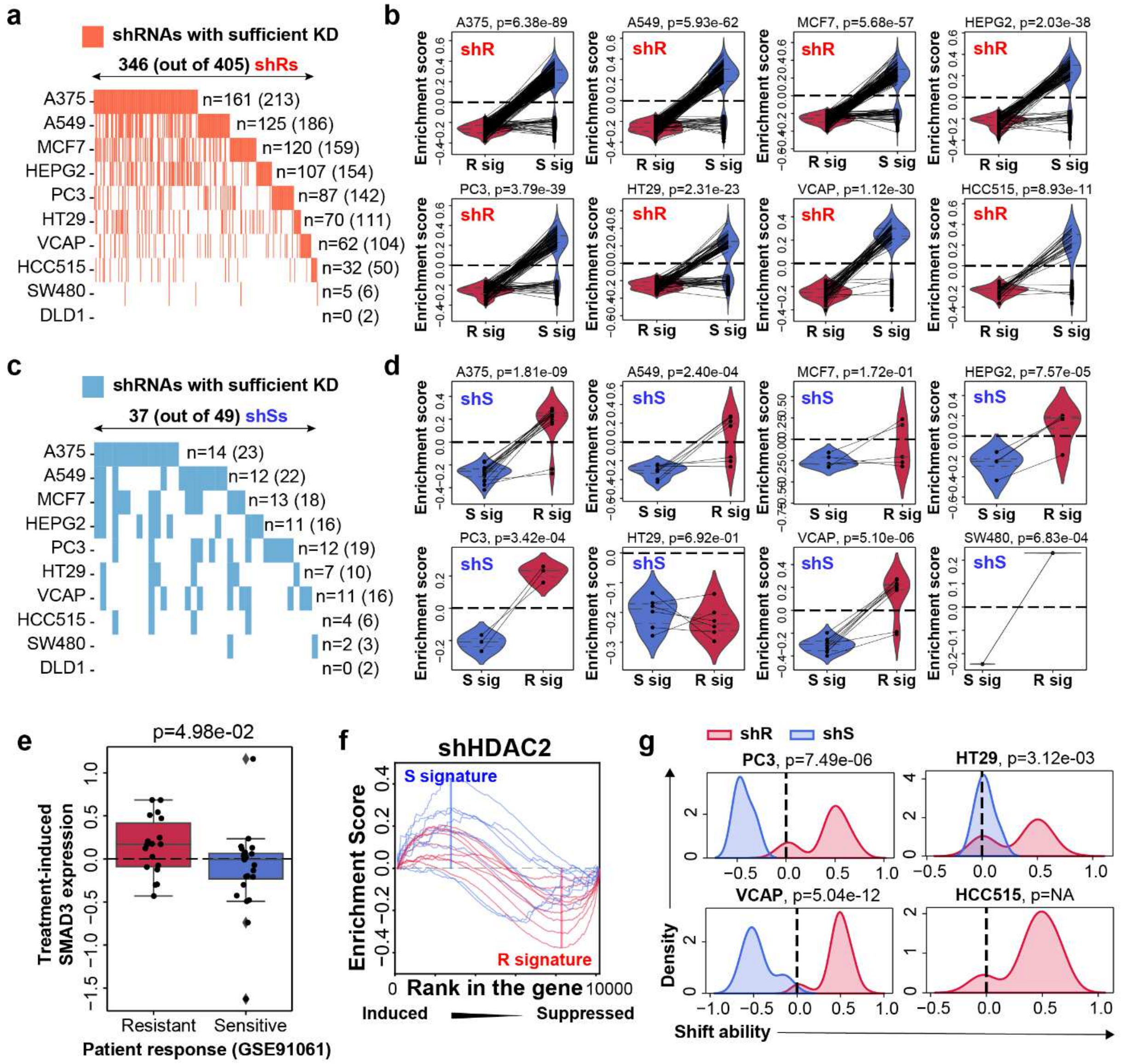

Extended Data Fig. 3 (Corresponding to Fig. 3) shRNAs targeting Resistance signature can induce Sensitivity signature gene expression in cancer cells.

a, Number of R-signatures targeting shRNAs with sufficient knockdown efficiency in different cell lines.

b, Enrichment score of R signature and S signature in the cell lines after R signature genes are being knocked down. Enrichment scores coming from the same experiment are connected by the lines. P values are given by paired t test.

c, Number of S-signatures targeting shRNAs with sufficient knockdown efficiency in different cell lines.

d, Enrichment score of R signature and S signature in the cell lines after S signature genes are being knocked down. Enrichment scores coming from the same experiment are connected by the lines. P values are given by paired t test.

e, Treatment-induced expression level of SMAD3 in anti-PD-1 treated patients (GSE91061). *P* value is given by student's t test.

f, Enrichment curves of R signature and S signature in HDAC2 knockdown cell lines.

g, Distribution of shift ability score of shRNAs targeting R signatures (shR) or S signatures (shS) across different cell lines. P values are given by two sample KS test.

Figure S4

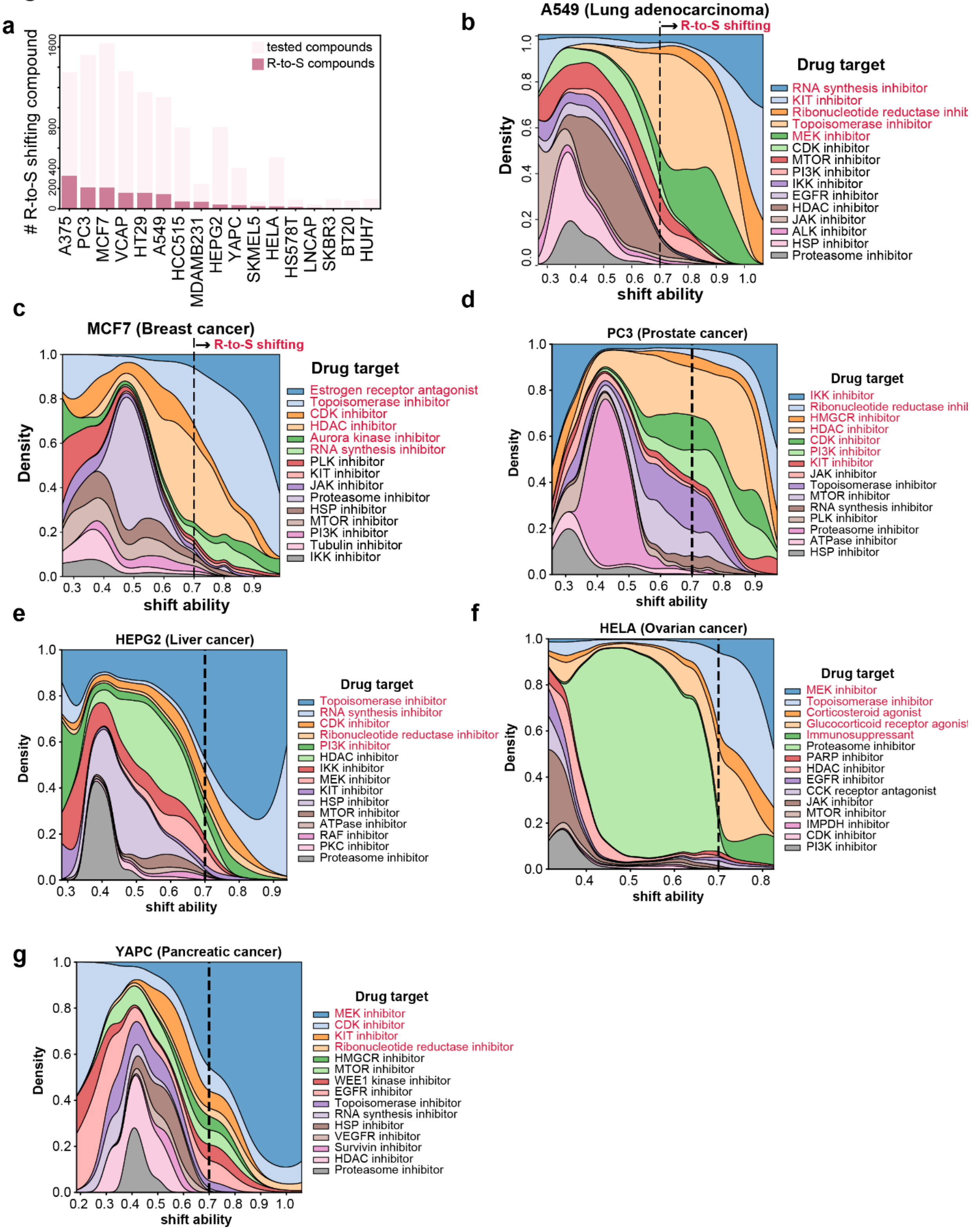

a
R-to-S shifting compound
tested compounds
R-to-S compounds
0
200
400
800
1600
A375
PC3
MCF7
VCAP
HT29
A549
HCC515
MDAMB231
HEPG2
YAPC
SKMEL5
HELA
HS578T
LNCAP
SKBR3
BT20
HUH7
b
A549 (Lung adenocarcinoma)
R-to-S shifting
Density
shift ability
Drug target
RNA synthesis inhibitor
KIT inhibitor
Ribonucleotide reductase inhit
Topoisomerase inhibitor
MEK inhibitor
CDK inhibitor
MTOR inhibitor
PI3K inhibitor
IKK inhibitor
EGFR inhibitor
HDAC inhibitor
JAK inhibitor
ALK inhibitor
HSP inhibitor
Proteasome inhibitor
c
MCF7 (Breast cancer)
R-to-S shifting
Density
shift ability
Drug target
Estrogen receptor antagonist
Topoisomerase inhibitor
CDK inhibitor
HDAC inhibitor
Aurora kinase inhibitor
RNA synthesis inhibitor
PLK inhibitor
KIT inhibitor
JAK inhibitor
Proteasome inhibitor
HSP inhibitor
MTOR inhibitor
PI3K inhibitor
Tubulin inhibitor
IKK inhibitor
d
PC3 (Prostate cancer)
Density
shift ability
Drug target
IKK inhibitor
Ribonucleotide reductase inhil
HMGCR inhibitor
HDAC inhibitor
CDK inhibitor
PI3K inhibitor
KIT inhibitor
JAK inhibitor
Topoisomerase inhibitor
MTOR inhibitor
RNA synthesis inhibitor
PLK inhibitor
Proteasome inhibitor
ATPase inhibitor
HSP inhibitor
e
HEPG2 (Liver cancer)
Density
shift ability
Drug target
Topoisomerase inhibitor
RNA synthesis inhibitor
CDK inhibitor
Ribonucleotide reductase inhibitor
PI3K inhibitor
HDAC inhibitor
IKK inhibitor
MEK inhibitor
KIT inhibitor
HSP inhibitor
MTOR inhibitor
ATPase inhibitor
RAF inhibitor
PKC inhibitor
Proteasome inhibitor
f
HELA (Ovarian cancer)
Density
shift ability
Drug target
MEK inhibitor
Topoisomerase inhibitor
Corticosteroid agonist
Glucocorticoid receptor agonist
Immunosuppressant
Proteasome inhibitor
PARP inhibitor
HDAC inhibitor
EGFR inhibitor
CCK receptor antagonist
JAK inhibitor
MTOR inhibitor
IMPDH inhibitor
CDK inhibitor
PI3K inhibitor
g
YAPC (Pancreatic cancer)
Density
shift ability
Drug target
MEK inhibitor
CDK inhibitor
KIT inhibitor
Ribonucleotide reductase inhibitor
HMGCR inhibitor
MTOR inhibitor
WEE1 kinase inhibitor
EGFR inhibitor
Topoisomerase inhibitor
RNA synthesis inhibitor
HSP inhibitor
VEGFR inhibitor
Survivin inhibitor
HDAC inhibitor
Proteasome inhibitor

Figure S4 cont'd

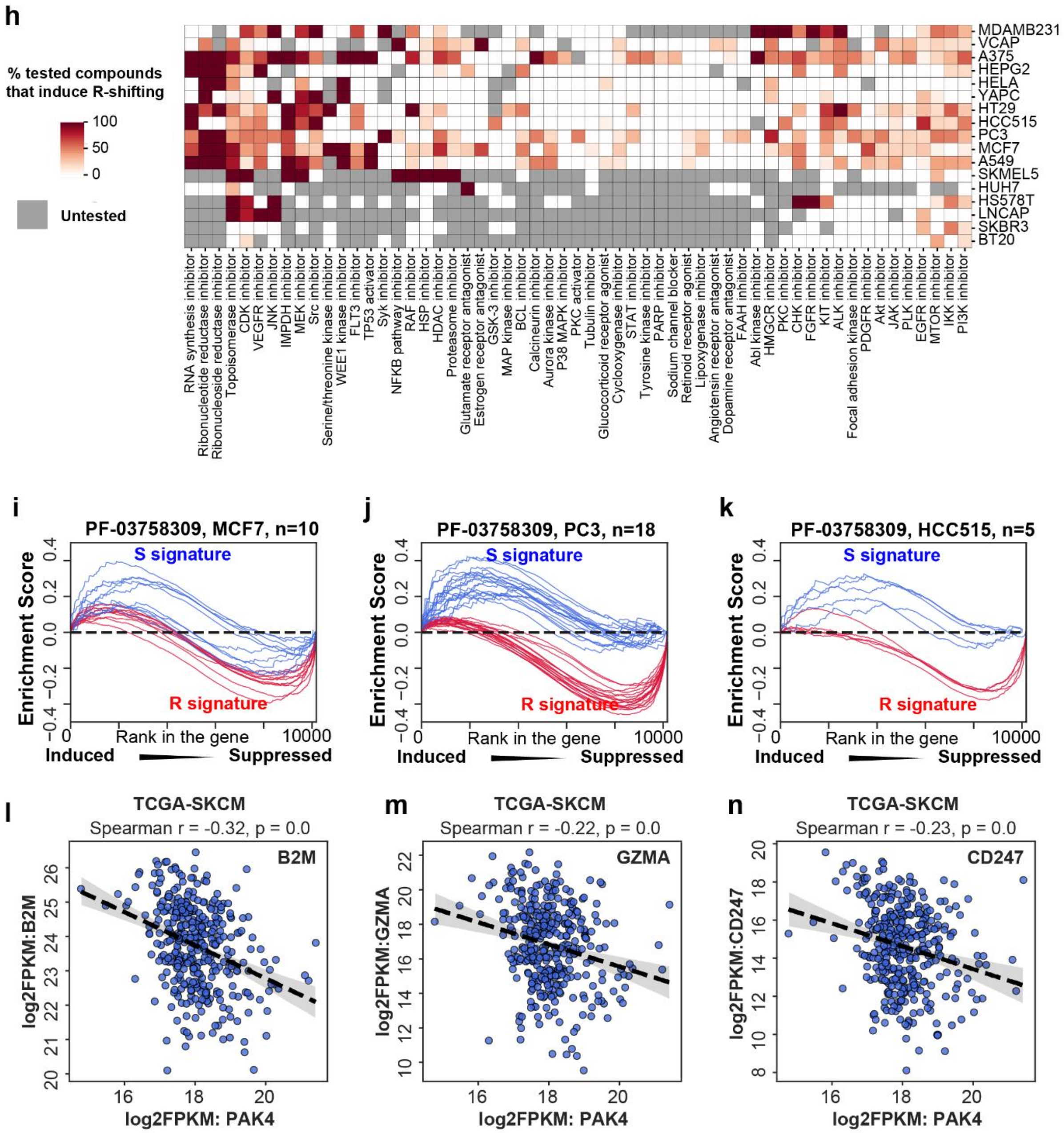

Extended Data Fig. 4 (Corresponding to Fig. 4) Shift ability analysis on compound-treated transcriptomes identified the landscape of chemo-immunotherapy synergism.

a, Number of R-to-S shifting compounds across different cell lines. Dark pink colored bars indicate the number of R-to-S shifting compounds. Light pink bars indicate the total number of tested compounds.

b, Stacked density plot of top R-to-S shifting drug targets in A549 lung adenocarcinoma cell line. X-axis indicates shift ability. Y-axis indicates density. Red-highlighted text indicates the major drug targets in significant R-to-S shifting range (shift ability >= 0.7).

c, Stacked density plot of top R-to-S shifting drug targets in MCF7 breast cancer cell line.

d, Stacked density plot of top R-to-S shifting drug targets in PC3 prostate cancer cell line.

e, Stacked density plot of top R-to-S shifting drug targets in HEPG2 liver cancer cell line.

f, Stacked density plot of top R-to-S shifting drug targets in HELA ovarian cancer cell line.

g, Stacked density plot of top R-to-S shifting drug targets in YAPC pancreatic cancer cell line.

h, Drug target enrichment of R-to-S shifting compounds across cancer cell lines. Untested cell lines are shaded by grey.

i to **k**, Enrichment curves of R signature and S signature in PAK4 inhibitor treated cell lines (MCF7, PC3 and HCC515).

l to **n**, Association between PAK4 gene expression and B2M, GZMA and CD247 in TCGA melanoma cohorts.

Figure S5

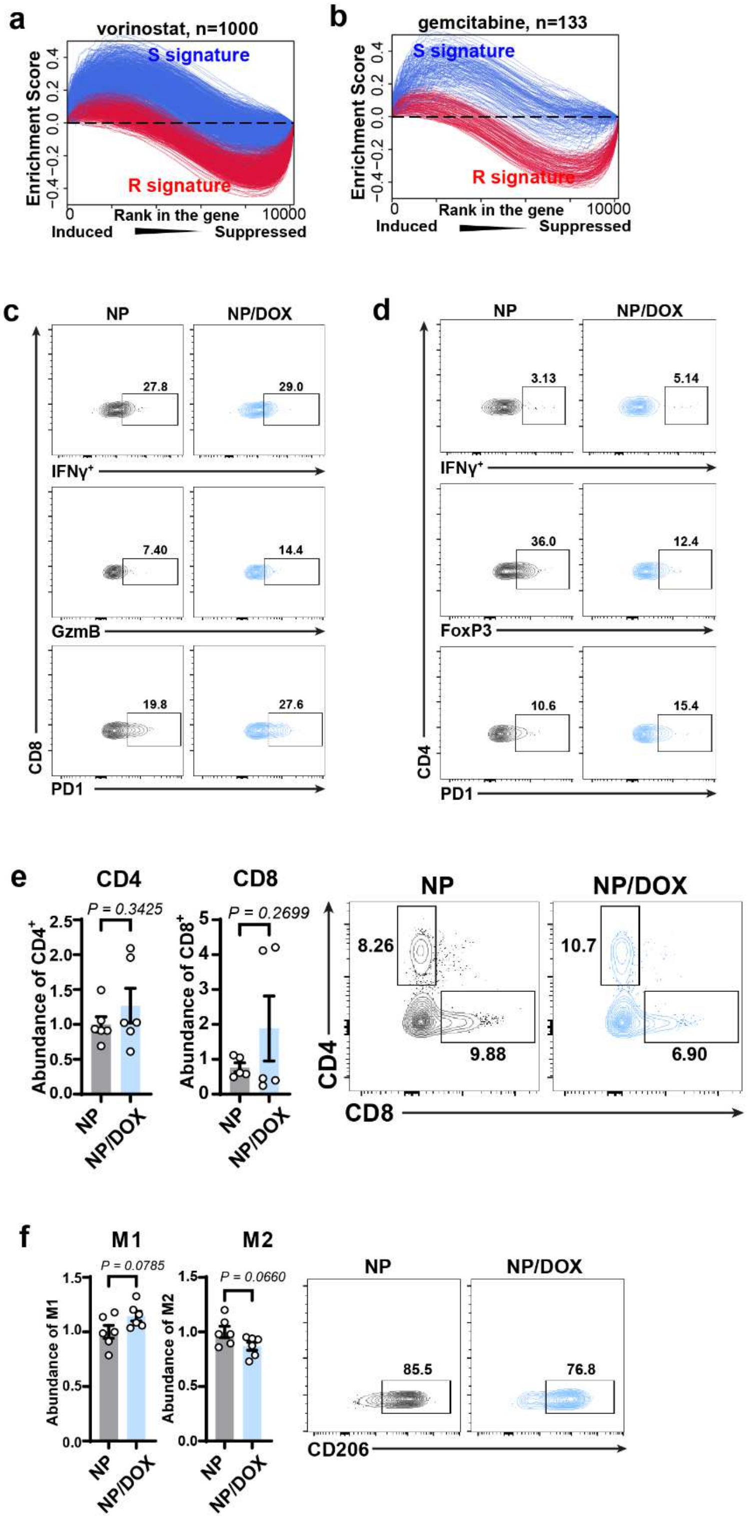

919

920 **Extended Data Fig. 5 (Corresponding to Fig. 5) Chemotherapy drugs can induce R-to-S**
921 **shifting in multiple cell lines.**

a, Enrichment curves of R signature and S signature in vorinostat treated cell lines.

b, Enrichment curves of R signature and S signature in gemcitabine treated cell lines.

c, Single-cell suspensions were prepared from CT26 tumor samples and subjected to flow cytometry analysis of $CD4^+$ subtype T cells ($CD4^+$ $IFN\gamma^+$ T cells, $FoxP3^+$ T cells, $CD4^+$ $PD\text{-}1^+$ T cells).

d, Single-cell suspensions were prepared from CT26 tumor samples and subjected to flow cytometry analysis of $CD8^+$ subtype T cells ($CD8^+$ $IFN\gamma^+$ T cells, $GzmB^+$ T cells, $CD8^+$ $PD\text{-}1^+$ T cells).

e, Single-cell suspensions were prepared from CT26 tumor samples and subjected to flow cytometry analysis of $CD4^+$ or $CD8^+$ cells.

f, Percentages of TAM population (M1 and M2) in CT26 colorectal tumor tissues.

Extended Data Figure 6

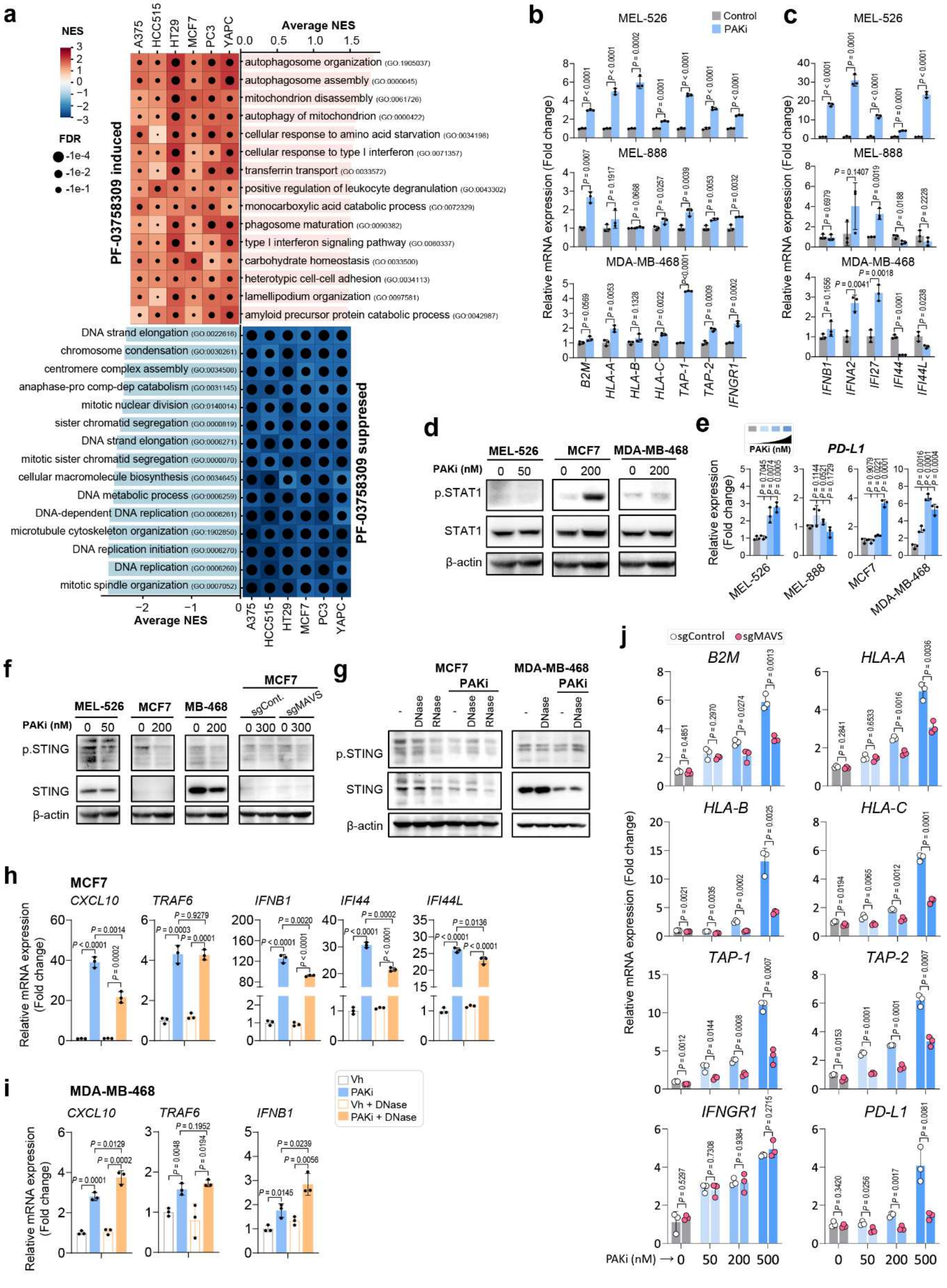

Extended Data Fig. 6 (Corresponding to Fig. 6) PAK4 inhibitor can induce immune response through autophagy-mtRNA-MAVS-CXCL10 axis in cancer cells.

a, Top 15 pathways that are induced (red) and suppressed (blue) by PF-03758309 treatment in multiple cancer cell lines. Heatmap showed the normalized enrichment score of gene ontology pathways in corresponding cancer cell lines. Bar plot on the side of the heatmap indicates the average normalized enrichment score across different cell lines.

b and **c**, qRT-PCR validation of antigen presenting and processing genes (d) and interferon stimulated genes (e) in cancer cells after 48 h of PAKi treatment. Concentration of PF-03758309 used: 50 nM for MEL-526, 200 nM for MEL-888, and MDA-MB-468. 0 nM or vehicle served as control. n = 3 technical replicates.

d, Immunoblotting analysis of STAT1 (**b**) and STING (**c**) in cancer cells after 48 h of treatment.

e, PAKi treatment induces PD-L1 expression in cancer cells (48h after treatment).

f, Immunoblotting analysis of STING in MEL-526, MCF7, MDA-MB-468 and MCF7 MAVS-KO cells were treated with PAKi for 48 h.

g, Immunoblotting analysis of STING in MCF7 and MDA-MB-468 cells were treated with PAKi (300 or 200 nM) for 48 h in presence of DNase or RNase.

h and **i**, qRT-PCR analysis of CXCL10 and ISG genes in MCF7 (**h**) and MDA-MB-468 (**i**) cells after 48 h of treatment in presence of DNase.

g, qRT-PCR analysis of antigen presenting and processing genes in MCF7 sgControl and sgMAVS cells following 48 h PAKi treatment.

Data in **b**, **c**, **e**, and **h-j** are presented as mean ± SD, n = 3 technical replicates, P values were generated using a two-tailed Student's t-test.

Tables

Supplementary Table 1. Treatment-induced expression change profiling can predict anti-PD-1 response in patients.

Supplementary Table 2. Genes involved in R and S signatures are highly correlated with patient prognosis and immune responses.

Supplementary Table 3. Genetic inhibition of genes in R and S signature can shift immunotherapy response phenotypes.

Supplementary Table 4. Shift ability analysis on compound-treated transcriptomes characterized chemo-immunotherapy synergism.

Supplementary Table 5. Integrating shift ability analysis on genetic and pharmacological inhibition identified novel compounds that can sensitize anti-PD-1 response.

Supplementary Table 6. Mechanism of chemo-immunotherapy synergisms.

Supplementary Table 7. qRT-PCR of antigen presentation genes after 48 h of treatment in cancer cells.

Supplementary Table 8. qRT-PCR analysis of type I interferon signaling genes after 48 h of treatment in cancer cells.
